# Network meta-analysis of HIF-prolyl hydroxylase inhibitors for anemia in dialysis-dependent and non-dialysis CKD: effects on hemoglobin, iron markers, and adverse clinical outcomes

**DOI:** 10.1186/s12882-025-04561-x

**Published:** 2025-11-14

**Authors:** Hasti Nasiri, Amirali Mirmazhari, Leila Mirzakhani, Parham Asgarian, Ali Gholibeigi, Tina Ghandali, Maryam Talebi Moghaddam, Mehdi Mohammadi, Mehdi Karimi, Atieh Makhlough

**Affiliations:** 1 School of Medicine, Mazandaran University of Medical Sciences, Sari, Islamic Republic of Iran; 2 Nephrology Department, Mazandaran University of Medical Sciences, Sari, Islamic Republic of Iran; 3 Department of Medical Biotechnology, School of Advanced Medical Technologies, Mazandaran University of Medical Sciences, Sari, Islamic Republic of Iran; 4 Faculty of Allied Medical Sciences, Mazandaran University of Medical Sciences, Sari, Islamic Republic of Iran; 5 Department of Internal Medicine, Gastrointestinal Cancer Research Center, Non-Communicable Diseases, Mazandaran University of Medical Sciences, Sari, Islamic Republic of Iran; 6 Department of Nephrology, Mazandaran University of Medical Sciences, Sari, Islamic Republic of Iran; 7 Neurology Research Center, School of Medicine, Mazandaran University of Medical Sciences, Sari, Islamic Republic of Iran; 8 Bu-Ali Sina Hospital, Mazandaran University of Medical Sciences, No. 20, Pasdaran Boulevard, Sari, 4815838477 Islamic Republic of Iran

**Keywords:** Adverse events, Anemia, Chronic kidney disease, Daprodustat, Dialysis, Hemoglobin, Hypoxia-inducible factor prolyl hydroxylase inhibitors, Network meta-analysis, Roxadustat, Transferrin saturation

## Abstract

HIF–prolyl hydroxylase inhibitors (HIF-PHIs) are oral alternatives to erythropoiesis-stimulating agents (ESAs) for anemia in chronic kidney disease (CKD). We performed a network meta-analysis comparing six HIF-PHIs (roxadustat, daprodustat, vadadustat, molidustat, enarodustat, desidustat) versus ESAs or placebo across hemoglobin efficacy, iron indices, and adverse events, with prespecified subgroup analyses by dialysis status. Forty-five randomized trials enrolling over 32,000 participants were analyzed using both frequentist and Bayesian frameworks with inconsistency checks. Outcomes included hemoglobin, ferritin, hepcidin, serum iron, total iron-binding capacity, and transferrin saturation; VEGF and lipid endpoints were not synthesized due to sparse, heterogeneous reporting. Across analyses, roxadustat and daprodustat increased hemoglobin more than ESA or placebo overall. Roxadustat tended to rank highest for hemoglobin, particularly in non-dialysis populations, whereas daprodustat showed advantages among dialysis-dependent patients and was associated with greater improvements in iron mobilization (lower hepcidin and ferritin, higher transferrin saturation). Estimates for desidustat and vadadustat were favorable but less precise, while evidence for enarodustat and molidustat was limited. Safety appeared class-neutral in aggregate; however, agent-specific patterns emerged—roxadustat showed higher rates of vascular occlusive events in some trials, daprodustat more gastrointestinal events, and molidustat a lower risk of hyperkalemia. Because SUCRA ranks reflect probability rather than effect magnitude, rankings were interpreted alongside absolute effects and study design. In sum, HIF-PHIs are not interchangeable; efficacy and safety vary by agent and dialysis status. Choice of therapy should consider inflammatory burden, iron handling, and adherence context. Head-to-head trials and real-world studies are needed to validate comparative findings and guide personalized use.

**Supplementary Information:**

The online version contains supplementary material available at 10.1186/s12882-025-04561-x.

## Clinical trial number

Not applicable.

## Introduction

Chronic kidney disease (CKD) is a growing global health concern, affecting nearly 700 million individuals worldwide. Its clinical and economic burden is expected to rise by 13.8 percent between 2022 and 2027 [1, 2]. Anemia is one of the most common complications of CKD, primarily caused by decreased erythropoietin production. It may appear in early stages, worsen over time [3], and be associated with reduced quality of life, increased hospitalization, and higher mortality rates [4, 5]. Erythropoiesis-stimulating agents (ESAs) have long been used to manage anemia in CKD [6]. These agents help reduce the need for blood transfusions and improve patient symptoms [7]. However, their effectiveness may be reduced in the presence of inflammation, iron deficiency, or uremic conditions, often requiring higher doses that increase the risk of adverse events. Additionally, their injectable administration poses practical challenges in routine care [8].

Hypoxia-inducible factor prolyl hydroxylase inhibitors (HIF-PHIs) have emerged as promising oral alternatives. By inhibiting prolyl hydroxylase domain enzymes, they stabilize HIF-α, which dimerizes with HIF-β to activate hypoxia-responsive genes [9, 10]. This mechanism enhances endogenous erythropoietin production and improves iron metabolism by lowering hepcidin levels and increasing the expression of iron transport proteins, thereby supporting erythropoiesis even under inflammatory conditions [11, 12].

Since 2015, multiple randomized controlled trials (RCTs) have investigated the efficacy and safety of individual HIF stabilizers. Several network meta-analyses)NMAs) [13–16] have compared these agents, often stratifying results by dialysis status. However, prior studies have generally been limited in drug coverage, outcomes assessed, or analytic approach.

In this network meta-analysis, we systematically compare roxadustat, daprodustat, vadadustat, molidustat, enarodustat, and desidustat against placebo or ESAs. We assess outcomes related to hemoglobin levels, iron parameters, and adverse events. Subgroup analyses based on dialysis status are included, using both frequentist and Bayesian frameworks to ensure robust and comprehensive evaluation.

## Method

### Protocol, registration, and eligibility criteria

This network meta-analysis followed a prespecified protocol registered in the International Prospective Register of Systematic Reviews (PROSPERO: CRD420251127908). The protocol defined objectives, eligibility criteria, outcomes, and analysis methods prior to data extraction. Eligible studies were RCTs enrolling adults (≥18 years) with CKD, regardless of dialysis status. Trials had to evaluate at least one HIF prolyl hydroxylase inhibitor (roxadustat, daprodustat, vadadustat, molidustat, enarodustat, or desidustat) compared to placebo or ESAs, and report at least one predefined outcome.

For continuous outcomes, trials were included only if post-treatment means and standard deviations (SDs) were available or could be derived. When only baseline and change-from-baseline data were reported, the final mean was calculated by adding the baseline mean to the mean change. The SD of the final value was estimated using the following formula, assuming a pre–post correlation coefficient of *r* = 0.5, in line with common practice in meta-analyses and recommended methodological guidelines [17, 18].

$${\rm{S}}{{\rm{D}}_{\left\{ {{\rm{final}}} \right\}}} = {\rm{ }}\sqrt {\left\{ {{\rm{SD}}_{\left\{ {{\rm{change}}} \right\}}^2 + {\rm{ SD}}_{\left\{ {{\rm{baseline}}} \right\}}^2 - {\rm{ }}2{\rm{ }} \cdot {\rm{r }} \cdot {\rm{S}}{{\rm{D}}_{\left\{ {{\rm{change}}} \right\}}} \cdot {\rm{S}}{{\rm{D}}_{\left\{ {{\rm{baseline}}} \right\}}}} \right\}} $$

For binary outcomes, event counts were required. Authors were contacted when essential data were missing. Exclusion criteria included non-randomized designs, pediatric populations, duplicates, inappropriate comparators, or insufficient data for analysis.

### Outcomes of interest

Outcomes were classified into three major domains: (1) Hematologic and iron metabolism biomarkers: hemoglobin (Hb), serum ferritin, hepcidin, serum iron, total iron-binding capacity (TIBC), transferrin saturation (TSAT). (2) **Adverse events** (binary outcomes): cardiovascular events (myocardial infarction, stroke, thrombosis, hypertension, CV death), metabolic/electrolyte disorders (hyperkalemia), gastrointestinal side effects (nausea, vomiting, diarrhea, constipation), fluid-related events (edema), ophthalmologic/neuro events (diabetic retinopathy, headache), interventional complications (blood transfusion, vascular occlusion/stenosis), and all-cause mortality. Outcomes were extracted as mean differences or standardized mean differences (SMDs) for continuous variables and odds ratios (ORs) for binary outcomes.

### Information sources and search strategy

We systematically searched PubMed, Embase, Scopus, Web of Science, and the Cochrane Central Register of Controlled Trials (CENTRAL) from inception to February 29, 2025. The search combined MeSH terms and free-text keywords structured with Boolean logic: (“HIF-PHI” OR “roxadustat” OR “daprodustat” OR “vadadustat” OR “molidustat” OR “enarodustat” OR “desidustat”) AND (“chronic kidney disease” OR “CKD” OR “renal insufficiency”) AND (“hemoglobin” OR “iron” OR “ferritin” OR “adverse events”). Detailed search strategies for each database are available in the Supplementary File. A final search update was conducted immediately before data analysis.

### Study selection

Two independent reviewers (M.M. and A.A.) screened titles, abstracts, and full texts based on predefined eligibility criteria. Cohen’s kappa coefficient was used to assess inter-rater agreement (threshold ≥0.80). Discrepancies were resolved through discussion or third-party adjudication (M.K.). The selection process followed PRISMA 2020 guidelines.

### Data extraction

Using a standardized Excel template, two reviewers independently extracted data on study characteristics (author, year, country, design, sample size, duration), population features (mean age, gender, dialysis status, baseline eGFR), intervention and comparator arms, outcome values (means and SDs or event counts), risk of bias assessments, and funding sources. Disagreements were resolved via consensus or author contact.

### Risk of bias assessment

Risk of bias was assessed independently by two reviewers (M.K and M.R) using the Cochrane Risk of Bias 2 (RoB 2) tool. It evaluates five domains: bias from the randomization process, deviations from intended interventions, missing data, outcome measurement, and selection of reported results. Each domain was rated as “low risk,” “some concerns,” or “high risk” based on RoB 2 guidelines. Discrepancies were resolved by discussion and consensus.

### Data synthesis, assessment of Heterogeneity/Inconsistency, and statistical environment

Initial pairwise meta-analyses were conducted for treatment comparisons with direct evidence using a random-effects model (DerSimonian and Laird). Statistical heterogeneity was assessed via Cochran’s Q, I^2^, and τ^2^. NMAs were then performed using both frequentist and Bayesian frameworks. The frequentist analysis employed the netmeta package in R to estimate treatment effects (mean differences, SMDs, or logORs) and 95% confidence intervals (CIs), with treatment rankings based on P-scores. For Bayesian analysis, we used BUGSnet interfaced with BUGS, implementing a Markov Chain Monte Carlo (MCMC) approach with 20,000 iterations, 5,000 burn-in, and a thinning interval of 5. Convergence was verified using trace plots and Gelman–Rubin diagnostics. Global inconsistency was tested via the design-by-treatment interaction model, while local inconsistency was assessed through loop-specific and node-splitting methods. Stratified analyses were conducted based on dialysis status (dialysis-dependent vs. non-dialysis-dependent) for all primary outcomes. All analyses were executed using R version 4.3.1, leveraging the following packages: netmeta, meta, BUGSnet, gemtc, rjags, igraph, ggplot2, and dmetar. Visualizations—including network graphs, SUCRA/P-score plots, and funnel plots—were customized for clarity and publication using aesthetic packages and ggplot-based enhancements.

## Result

### Characteristics of included studies

**Fig. 1:**
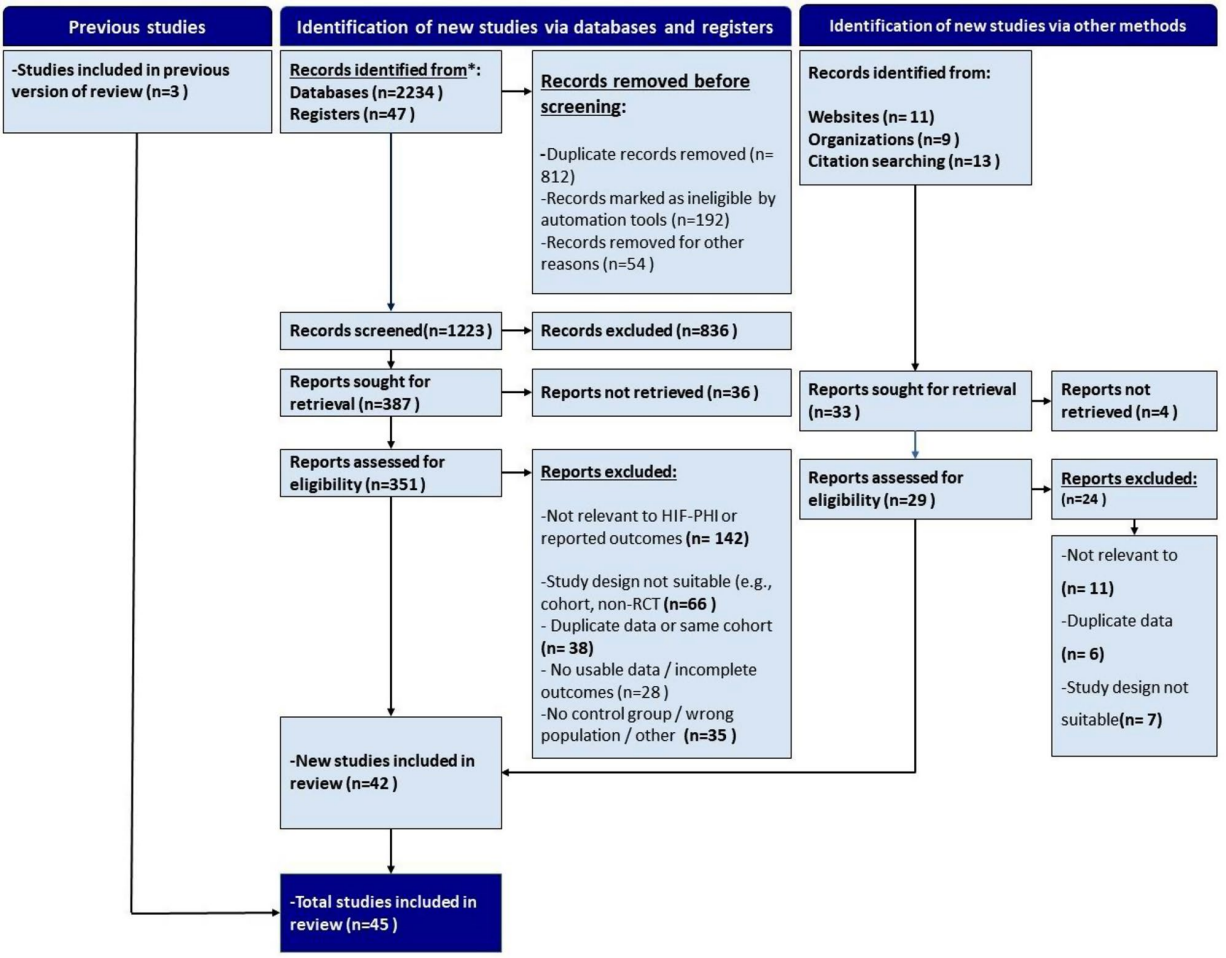
PRISMA flow diagram illustrating the study selection process

- This NMA incorporated data from 45 RCTs, encompassing over 32,000 participants with CKD, both dialysis-dependent and non-dialysis-dependent (Fig. 1). These trials were conducted across diverse geographic regions, including Asia, North America, and Europe, between 2015 and 2023. The interventions evaluated comprised six HIF-PHIs—roxadustat, daprodustat, vadadustat, molidustat, enarodustat, and desidustat—compared against ESAs or placebo. Most studies applied a parallel-group design with treatment durations ranging from 4 to 104 weeks. Baseline characteristics varied across studies. Mean participant age across the included trials ranged from approximately 48 to over 72 years, reflecting the demographic diversity of the CKD population. Several studies reported multiple treatment arms stratified by CKD stage or dialysis dependency. In addition to hemoglobin-related outcomes, a broad spectrum of iron metabolism markers and safety-related endpoints were reported, providing a rich basis for network synthesis. A detailed overview of study-level characteristics is presented in Table 1.

**Table 1:** Characteristics of the randomized controlled trials (RCTs) included in the network meta-analysis evaluating the efficacy and safety of hypoxia-inducible factor prolyl hydroxylase inhibitors (HIF-PHIs) in patients with chronic kidney disease

Author (Year)	Country	Study design	Sample size (int/cont)	Mean Age	CKD status	HIF-PHI type	Control treatment	Follow up	Specified biochemical outcomes	Adverse event-related outcomes
Besarab 2015 [19]	USA	RCT	88/28	65.8	NDD-CKD	Roxadustat	Placebo	4	CV Death/Diarrhea/Edema/Fatigue/Headache/Hyperkalemia/ Transfusion	Hb/Serum ferritin/Serum iron/TIBC/TSAT/
Provenzano 2016 [20]	USA	RCT	108/36	56.7	DD-CKD	Roxadustat	Epoetin alfa	19	Cancer/CV Death/Edema/Hyperkalemia/Myocardial infarction/Nausea/Peripheral arterial event/Vomiting	Hb/Hepcidin/Serum ferritin/Serum iron/TIBC/TSAT/
Chen 2017 [21]	China	RCT	61/30*** 74/22	60.2	NDD/DD-CKD	Roxadustat	Placebo, epoetin alfa	6	Abdominal pain/CV Death/Death/Diarrhea/Headache/Hyperkalemia/Hypertension/Nausea/Transfusion/Vomiting	Hb/Hepcidin/Serum ferritin/Serum iron/TIBC/TSAT/LDL/HDL/
Akizawa 2019 (A) [22]	Japan	RCT	80/27	63.8	NDD-CKD	Roxadustat	Placebo	24	CV Death/Death/Diarrhea/Nausea/Stroke	Hb/Hepcidin/Serum ferritin/TIBC/TSAT/
Chen 2019 (A) [23]	China	RCT	101/51	54.2	NDD-CKD	Roxadustat	Placebo	8	CV Death/Diarrhea/Edema/Hyperkalemia/Hypertension	Hb/Serum ferritin/Serum iron/TIBC/TSAT/
Chen 2019 (B) [24]	China	RCT	204/100	49	DD-CKD	Roxadustat	Epoetin alfa	27	CV Death/Death/Hyperkalemia/Hypertension/Occlusion Stenosis/Peripheral arterial event/Thrombosis/Vomiting	Hb/Serum ferritin/Serum iron/TIBC/TSAT
Akizawa 2020 (A) [25]	Japan	RCT	150/151	64.7	DD-CKD	Roxadustat	Darbepoetin alfa	24	Abdominal pain/Cancer/CV Death/Death/Diabetic retinopathy/Hyperkalemia/Occlusion Stenosis/Myocardial infarction/Vomiting	Hb/Serum ferritin/Serum iron/TIBC/TSAT/
Akizawa 2021 (A) [26]	Japan	RCT	201/131	69.5	NDD-CKD	Roxadustat	Darbepoetin alfa	24	CV Death/Death/Edema/Hyperkalemia	Hb/Hepcidin/Serum ferritin/Serum iron/TIBC/TSAT
Barratt 2021 [27]	Multicenter trial	RCT	323/293	66.3	NDD-CKD	Roxadustat	Darbepoetin alfa	104	CV Death/Death/Edema/Heart failure/hospitalization/Hyperkalemia/Hypertension/Myocardial infarction/Stroke/Peripheral arterial event/Thrombosis/Transfusion	Hb/Serum ferritin/Serum iron/TSAT/LDL
Fishbane 2021 [28]	Multicenter trial	RCT	1384/1377	61.2	NDD-CKD	Roxadustat	Placebo	52	Constipation/Diarrhea/Edema/Headache/Hyperkalemia/Hypertension/Nausea/Thrombosis/Transfusion/Vomiting	Hb/Hepcidin/Serum ferritin/Serum iron/TIBC/TSAT/LDL
Hou 2021 [29]	China	RCT	86/43	48.1	DD-CKD	Roxadustat	ESAs	24	Constipation/CV Death/Death/Headache/Hyperkalemia/Hypertension/Myocardial infarction/Vomiting	Hb/Hepcidin/Serum ferritin/TSAT
Provenzano 2021 [30]	Multicenter trial	RCT	522/521	54	DD	Roxadustat	Epoetin alfa	52	Death/Edema/Hyperkalemia/Occlusion Stenosis/Myocardial infarction/Transfusion	Hb/Hepcidin/Serum ferritin/Serum iron/TIBC/TSAT
Holdstock 2016 [31]	Multicenter trial	RCT	54/18*** 62/20	62.8	DD/NDD	Daprodustat	Placebo	4		Hb/Hepcidin/Serum ferritin/Serum iron/TIBC/TSAT
Akizawa 2017 [32]	Japan	RCT	78/19	62.4	DD	Daprodustat	Placebo	4	CV Death/Occlusion Stenosis/Thrombosis	Hb/Hepcidin/Serum ferritin/TIBC/TSAT
Singh 2021 (A) [33]	Multicenter trial	RCT	1937/1935	67	NDD	Daprodustat	Darbepoetin alfa	52	CV Death/Death/Edema/Heart failure hospitalization/Hyperkalemia/Myocardial infarction/Stroke/Thrombosis/Transfusion	Hb/Hepcidin/Serum ferritin/TSAT
Singh 2021 (B) [34]	Multicenter trial	RCT	1487/1477	58.5	DD	Daprodustat	ESAs	52	CV Death/Death/Edema/Heart failure hospitalization/Hyperkalemia/Myocardial infarction/Stroke/Thrombosis/Transfusion	Hb/Hepcidin/Serum ferritin/TSAT
Pergola 2016 [35]	USA	RCT	138/72	66.4	NDD	Vadadustat	Placebo	20	Constipation/Death/Diarrhea/Edema/Fatigue/Headache/Hyperkalemia/Hypertension/Nausea/Transfusion	Hb/Hepcidin/Serum ferritin/TIBC/
Martin 2017 [36]	USA	RCT	72/19	65.5	NDD	Vadadustat	Placebo	6		Hb/Hepcidin/Serum ferritin/TIBC/
Nangaku 2020 [37]	Japan	RCT	37/14*** 44/14	66.8	NDD/DD	Vadadustat	Placebo	6	CV Death/Occlusion Stenosis/Transfusion	Hb
Chertow 2020 [38]	Multicenter trial	RCT	1741/1735	66	NDD	Vadadustat	Darbepoetin alfa	52	Death/Diabetic retinopathy/Diarrhea/Edema/Fatigue/Hyperkalemia/Hypertension/Myocardial infarction/Nausea/Thrombosis	Hb
Eckardt 2021 [39]	Multicenter trial	RCT	1958/1965	57.9	DD	Vadadustat	Darbepoetin alfa	52	Death/Diarrhea/Hyperkalemia/Hypertension/Nausea/Thrombosis/Vomiting	Hb/Hepcidin/Serum ferritin/TSAT/
Nangaku 2021 (B) [40]	Japan	RCT	151/153	72	NDD	Vadadustat	Darbepoetin alfa	52	Cancer/Constipation/Death/Diabetic retinopathy/Diarrhea/Edema/Heart failure hospitalization/Hyperkalemia/Hypertension/Myocardial infarction/Thrombosis/Vomiting	Hb/Hepcidin/Serum ferritin/TSAT/
Nangaku 2021 (C) [41]	Japan	RCT	162/161	65.5	DD	Vadadustat	Darbepoetin alfa	52	Cancer/Constipation/Death/Diabetic retinopathy/Diarrhea/Edema/Headache/Hyperkalemia/Occlusion Stenosis/Nausea/Thrombosis/Vomiting	Hb/Serum ferritin/TSAT/
Akizawa 2019 B [42]	Japan	RCT	60/22	61.9	DD	Enarodustat	Placebo	30		Hb/Serum ferritin/TIBC/TSAT
Akizawa 2021 B [43]	Japan	RCT	107/109	69.7	NDD	Enarodustat	Darbepoetin alfa	24	Cancer/CV Death/Death/Diarrhea/Edema/Hypertension/Thrombosis	Hb/Serum ferritin/Serum iron/TIBC/TSAT
Akizawa 2021 (C) [44]	Japan	RCT	86/86	64	DD	Enarodustat	Darbepoetin alfa	24	Cancer/CV Death/Death/Diabetic retinopathy/Edema/Hypertension/Occlusion Stenosis/Myocardial infarction/Thrombosis/Vomiting	Hb/Serum ferritin/Serum iron/TIBC/TSAT/
Macdougall 2019 [45]	Multicenter trial	RCT	350/94	62.6	NDD/ DD	Molidustat	rHuEPO	16	Constipation/CV Death/Death/Diarrhea/Edema/Hyperkalemia/Hypertension/Myocardial infarction/Nausea/Stroke/Peripheral arterial event/Transfusion/Vomiting	Hb/Serum ferritin/Serum iron/TIBC/TSAT
Akizawa 2021 (D) [46]	Japan	RCT	153/76	65.7	DD	Molidustat	Darbepoetin alfa	36	Abdominal pain/Cancer/Constipation/CV Death/Death/Diabetic retinopathy/Diarrhea/Headache/Hyperkalemia/Occlusion Stenosis/Myocardial infarction/Nausea/Vomiting	Hb/Serum ferritin/Serum iron/TIBC/TSAT
Holdstock 2019 [47]	USA	RCT	156/79	66/5	NDD	Daprodustat	rHuEPO	16	Death/Diarrhea /Heart failure hospitalization/Myocardial infarction/Nausea/Stroke	Hb/Hepcidin/Serum ferritin/Serum iron/TIBC/TSAT
Meadowcroft 2019 [48]	Multicenter trial	RCT	171/39	59.7	DD	Daprodustat	rHuEPO	24	Diarrhea/Heart failure hospitalization/Headache/Hyperkalemia/Hypertension/Myocardial infarction/Nausea/Stroke	Hb/Hepcidin/Serum ferritin/Serum iron/TIBC/TSAT
Akizawa 2020 (B) [49]	Japan	RCT	136/135	64	DD	Daprodustat	Darbepoetin alfa	52	Constipation/CV Death/Death/Diabetic retinopathy/Diarrhea/Edema/Headache/Hyperkalemia/Hypertension/Occlusion Stenosis/Nausea/Vomiting	Hb/Hepcidin/Serum ferritin/Serum iron/TIBC/TSAT
Nangaku 2021 (A) [50]	Multicenter trial	RCT	149/150	70	NDD	Daprodustat	Epoetin beta pegol	52	Cancer/Constipation/CV Death/Death/Diabetic retinopathy/Diarrhea/Edema/Hyperkalemia/Hypertension	Hb/Hepcidin/Serum ferritin/Serum iron/TIBC/TSAT
Yamamoto 2021 (A) [51]	Japan	RCT	82/82	70.7	NDD	Molidustat	Darbepoetin alfa	52	Constipation/CV Death/Death/Diabetic retinopathy/Diarrhea/Edema/Hyperkalemia/Hypertension/Myocardial infarction/Transfusion	Hb/Serum ferritin/Serum iron/TIBC/TSAT
Yamamoto 2021 (B) [52]	Japan	RCT	82/80	71.7	NDD	Molidustat	Darbepoetin alfa	52	Constipation/CV Death/Death/Diarrhea/Hyperkalemia/Hypertension/Myocardial infarction/Nausea/Transfusion	Hb/Serum ferritin/Serum iron/TIBC/TSAT
Parmar 2019 [53]	India	RCT	87/30	48.1	NDD	Desidustat	Placebo	6	Abdominal pain/Headache/Vomiting	Hb/Serum iron/TIBC/TSAT
Agrawal 2022 [54]	India and Sri Lanka	RCT	294/294	52.77	NDD	Desidustat	Darbepoetin alfa	24	Vomiting/Myocardial infarction/Hypertension/Edema/Death/CV Death	Hb/VEGF/HDL
Gang 2022 [55]	India	RCT	196/196	50.96	DD	Desidustat	Epoetin alfa	24	Vomiting/Thrombosis/Nausea/Hypertension/Hyperkalemia/Headache/Edema/Diarrhea/Death	Hb/Hepcidin
Singh 2022 [56]	Multicenter trial	RCT	157/155	52	DD	Daprodustat	Darbepoetin	52	Stroke/Myocardial infarction/Hypertension/Headache/Diarrhea/Thrombosis/Hyperkalemia/Edema Death/CV Death/	Hb/Serum iron/TIBC/TSAT/Hepcidin
Akizawa 2019 (D1,2,4) [57]	Multicenter trial	RCT	D1: 101/20 D2: 92/32 D4: 157/42	D1: 68.4 D2: 67.9 D4: 59.3	**D1**: NDD **D2**: NDD **D4**: DD	D1: Molidustat D2: Molidustat D4: Molidustat	D1: Placebo D2:Darbepoetin D4: Epoetin	16		Hb/Serum ferritin/Hepcidin/Serum iron/TIBC/TSAT
Charytan 2021 [58]	USA	RCT	370/371	58	DD	Roxadustat	Epoetin alfa	52	Death/Hyperkalemia/Myocardial infarction/Thrombosis/Transfusion	Hb/Hepcidin/Serum ferritin/Serum iron/TIBC/TSAT
Coyne 2021 [59]	USA	RCT	616/306	64.9	NDD	Roxadustat	Placebo	52	Abdominal pain/Constipation/Diarrhea/Edema/Headache/Hyperkalemia/Hypertension/Nausea/Transfusion/Vomiting	Hb/Hepcidin/Serum ferritin/Serum iron/TIBC/TSAT
Csiky 2021 [60]	Multicenter trial	RCT	414/420	61.4	DD	Roxadustat	ESAs	52	Death/Hyperkalemia/Thrombosis/Transfusion	Hb/Hepcidin/Serum ferritin/TSAT
Shutov 2021 [61]	Multicenter trial	RCT	391/203	62.5	NDD	Roxadustat	Placebo	52	Diarrhea/Edema/Headache/Hyperkalemia/Hypertension/Nausea/Stroke/Thrombosis	Hb/Hepcidin/Serum ferritin/TSAT
Brigandi 2016 [62]	Multicenter trial	RCT	70/37	56.14	DD/NDD	Daprodustat	Placebo	4	Abdominal pain/CV Death/Diarrhea/Headache/Nausea/Vomiting	Hb/Hepcidin
Bailey 2019 [63]	Multicenter trial	RCT	79/18	63.4	DD	Daprodustat	Placebo	4	Diarrhea/Occlusion Stenosis	Hb/Serum iron/TIBC/TSAT
Akizawa 2019 (D3,5) [64]	Multicenter trial	RCT	D3: 118/42 D5: 57/30	D3: 69 D5: 60	D3: NDD D5: DD	D3: Molidustat D5: Molidustat	D3:Darbepoetin D5:Darbepoetin	52	Abdominal pain/Diarrhea/Hypertension	Hb/Serum ferritin/

Abbreviations: RCT – randomized controlled trial; CKD – chronic kidney disease; NDD – non-dialysis-dependent; DD – dialysis-dependent; HIF-PHI – hypoxia-inducible factor prolyl hydroxylase inhibitor; ESA – erythropoiesis-stimulating agent; Hb – hemoglobin; TSAT – transferrin saturation; TIBC – total iron-binding capacity

### Risk of bias

**Fig. 2:**
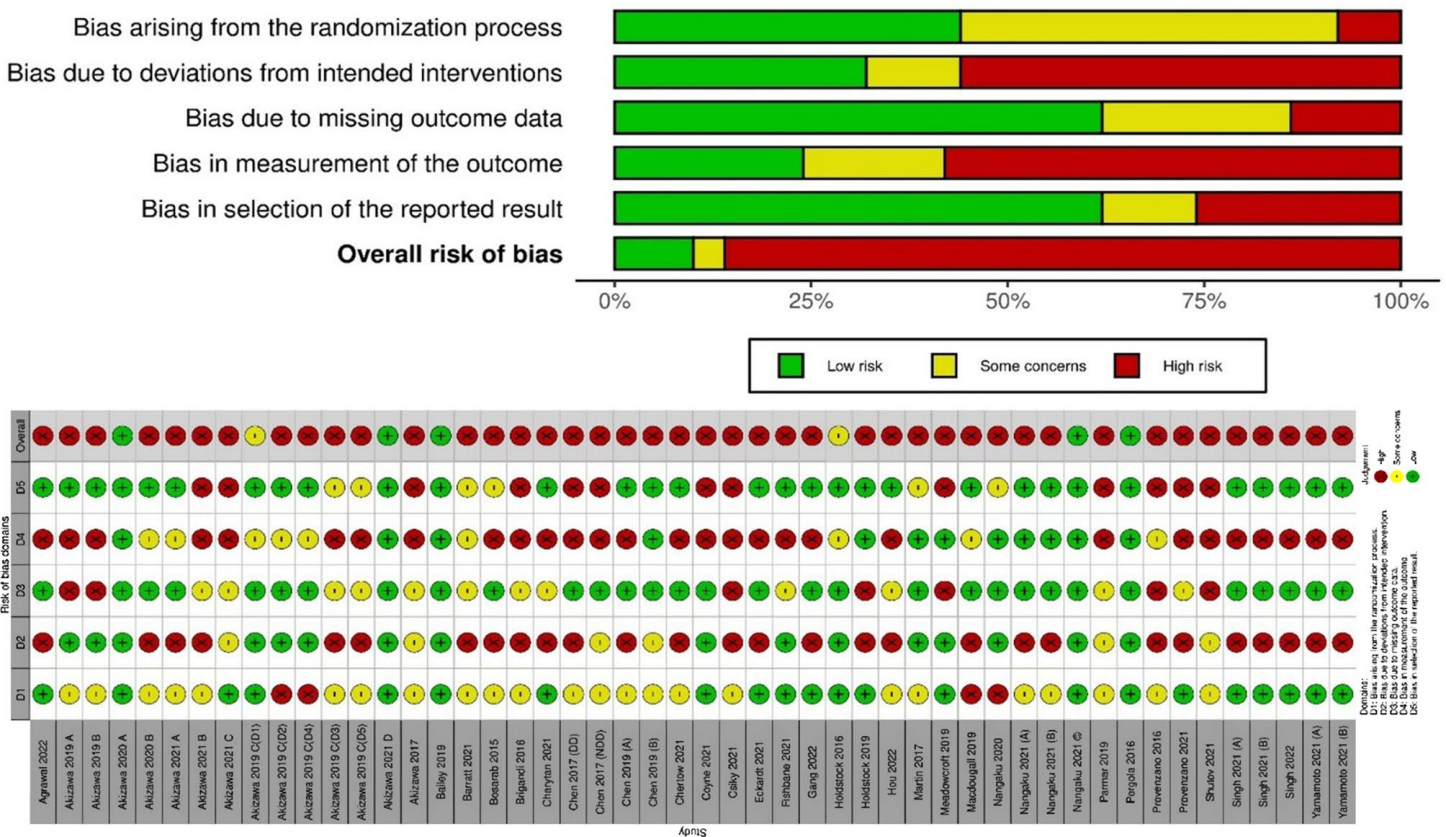
Risk of bias assessment for included studies

- Risk of bias was assessed using the RoB 2 tool across five standard domains in all included trials. Most studies showed low risk in the measurement of outcomes and in the selection of the reported results. However, over 50% of trials raised high risk in domains related to the randomization process and missing outcome data. Bias due to deviations from intended interventions was also frequently rated as high or with some concerns. Only a small proportion of trials, approximately 18%, were judged as low risk overall. The detailed assessment of individual domains is illustrated in Fig. 2.

#### Hemoglobin

**Fig. 3:**
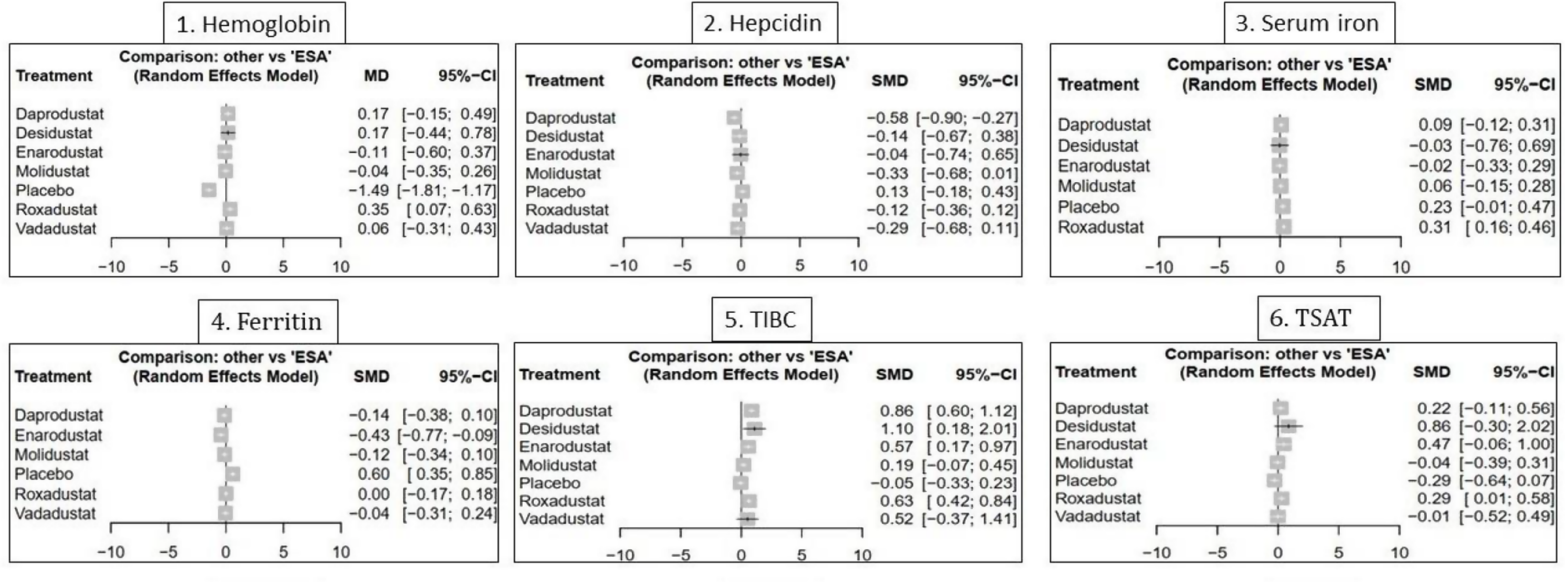
Forest plots demonstrating the comparative effects of HIF-PHI agents versus erythropoiesis-stimulating agents (ESAs) on laboratory outcomes. The results are presented for nine biomarkers (**1**): Hemoglobin (**2**), Hepcidin (**3**), Serum iron (**4**), Ferritin (**5**), TIBC (**6**), TSAT. Effect sizes are reported as mean differences (MD) or standardized mean differences (SMD) with 95% CIs using a random-effects model

In this NMA, Hb response was evaluated across 45 RCTs comparing six HIF-PHIs—roxadustat, daprodustat, vadadustat, molidustat, enarodustat, and desidustat—with ESAs or placebo in over 32,000 CKD patients. The treatment network demonstrated strong connectivity, particularly around roxadustat (*n* = 9) and vadadustat (*n* = 5), ensuring reliable estimation of both direct and indirect effects. Node-splitting analysis revealed no statistically significant inconsistencies between treatment comparisons (sTable 1 In Supplementary File). (Fig. 3)

**Fig. 4:**
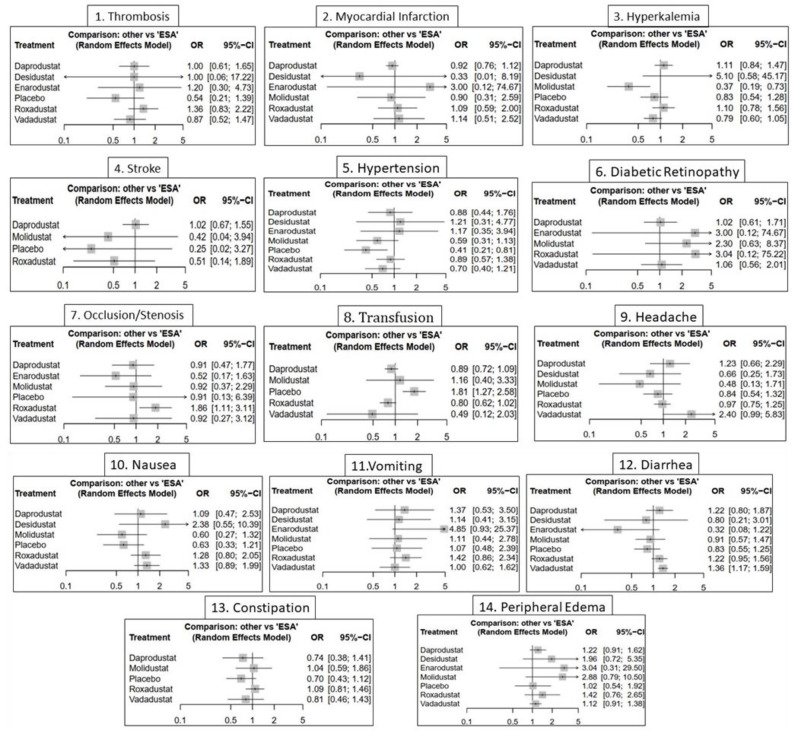
Forest plots illustrating the comparative safety profile of HIF-PHI agents versus erythropoiesis-stimulating agents (ESAs) across 14 adverse outcomes: (**1**) Thrombosis, (**2**) Myocardial infarction, (**3**) Hyperkalemia, (**4**) Stroke, (**5**) Hypertension, (**6**) Diabetic retinopathy, (**7**) Occlusion/Stenosis, (**8**) Transfusion, (**9**) Headache, (**10**) Nausea, (**11**) Vomiting, (**12**) Diarrhea, (**13**) Constipation, and (**14**) Peripheral edema. Results are presented as odds ratios (or) with 95% CIs using a random-effects model

**Fig. 5:**
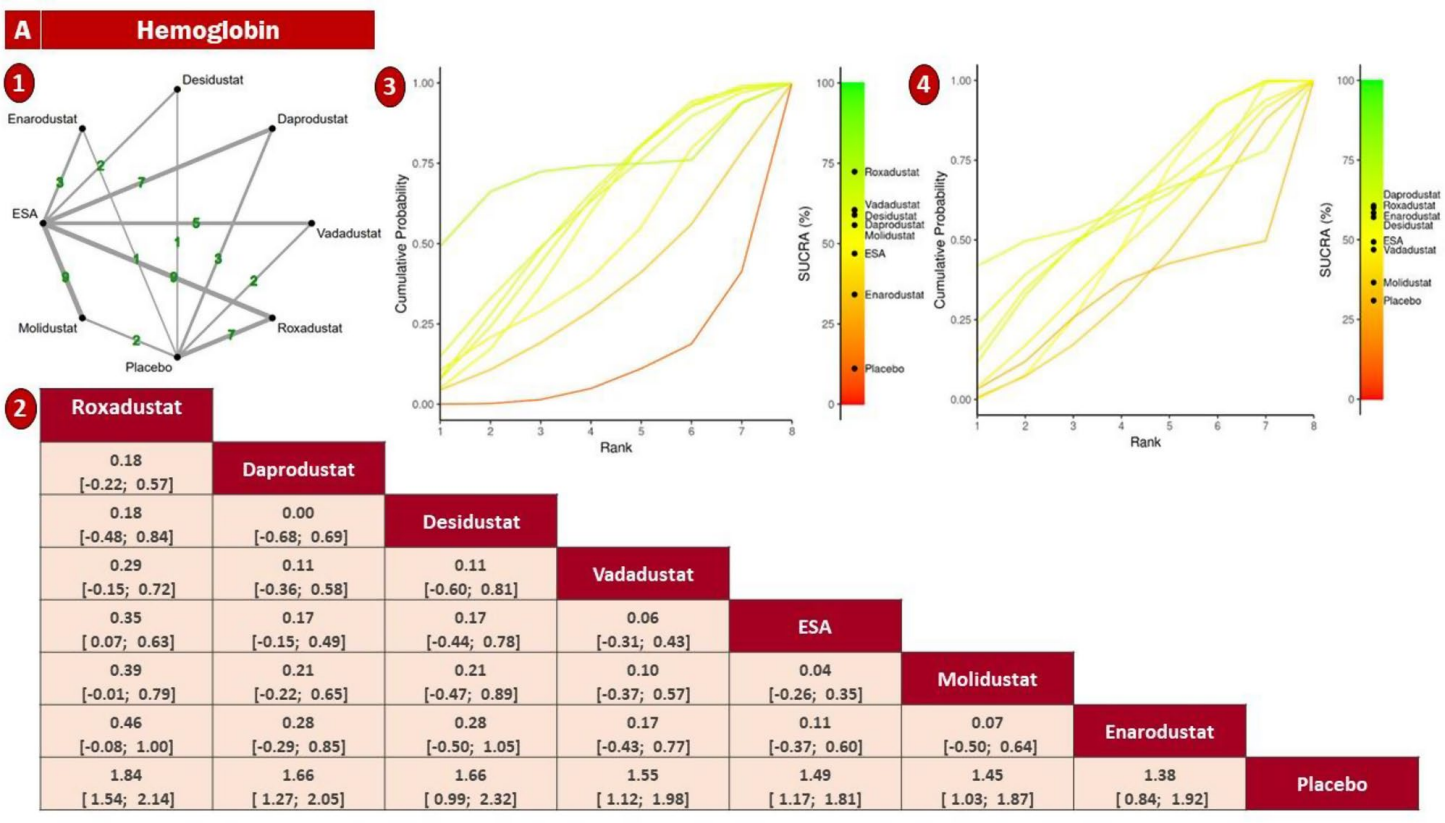
Network geometry, league table (overall), and SUCRA-based ranking of treatments for hemoglobin. Panels show: (**1**) Network plot of treatment comparisons; (**2**) League table of relative treatment effects; (**3**) Sucra rankings in non-dialysis patients; (**4**) sucra rankings in dialysis patients

Pairwise analyses indicated that roxadustat significantly increased hemoglobin compared to ESA (mean difference = +0.38 g/dL; 95% CI: +0.06 to +0.70) and placebo (+1.79 g/dL; 95% CI: +1.42 to +2.16), marking it as the most effective agent. daprodustat showed a comparable benefit over placebo (+1.61 g/dL; 95% CI: +1.01 to +2.21), but not against ESA (See Fig. 4). Other agents, such as vadadustat and desidustat, presented favorable trends but lacked statistical significance, as their confidence intervals crossed the null. Estimates for enarodustat and molidustat were highly imprecise due to sparse data (Fig. 5).

Treatment hierarchy based on SUCRA rankings placed roxadustat firmly at the top, followed by daprodustat, desidustat, and vadadustat. ESA and placebo consistently occupied the lowest ranks. This ranking was coherent with the pairwise findings and remained robust in sensitivity analyses (sFig. 9 In Supplementary File).

Subgroup analysis by dialysis status revealed distinct patterns. In non-dialysis-dependent (NDD) patients, roxadustat demonstrated superior performance, suggesting higher efficacy in early-stage CKD. Conversely, daprodustat was more effective in dialysis-dependent (DD) patients (Fig. 5).

#### Hepcidin

**Fig. 6:**
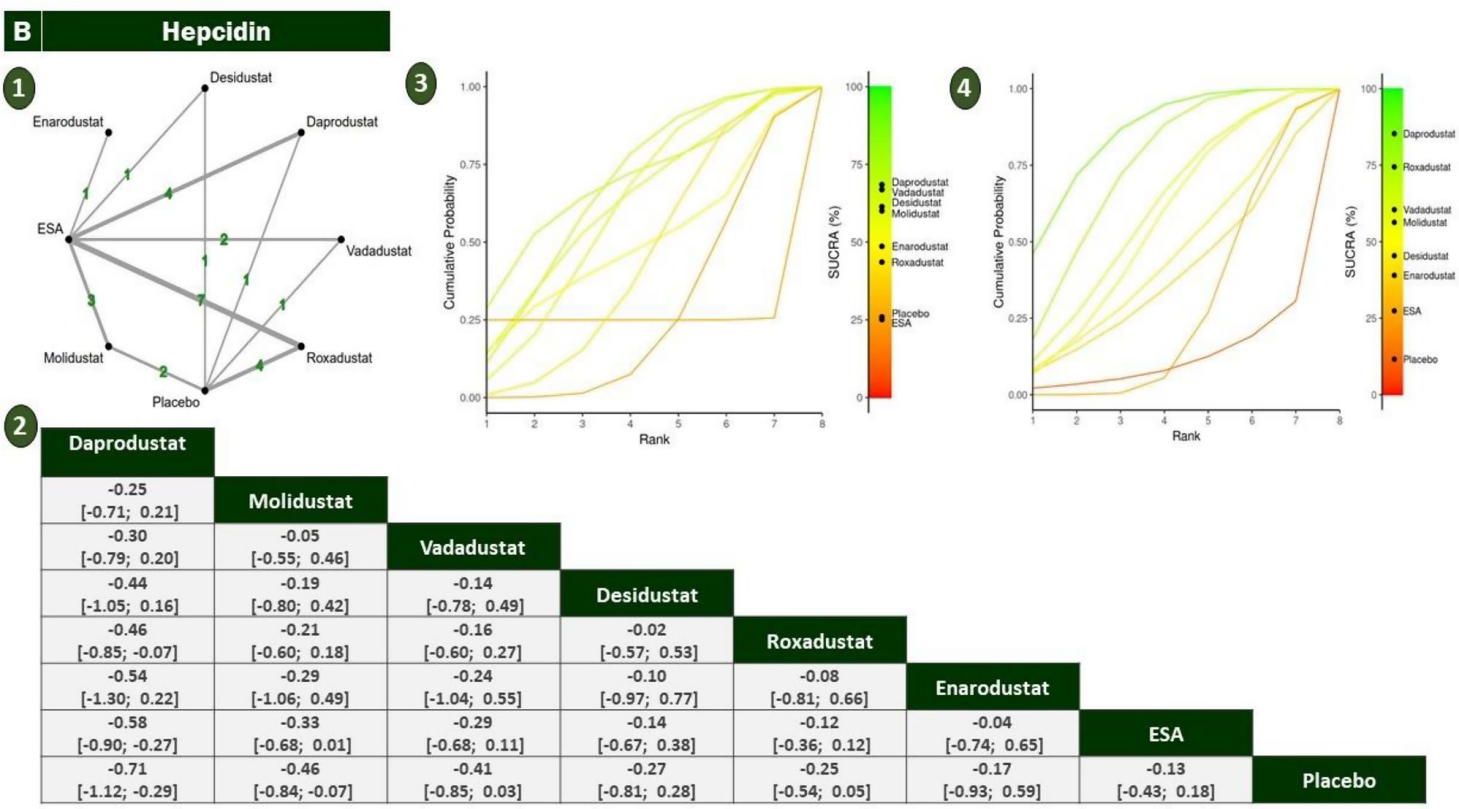
Network geometry, league table (overall), and SUCRA-based ranking of treatments for hepcidin. Panels show: (**1**) Network plot of treatment comparisons; (**2**) League table of relative treatment effects; (**3**) sucra rankings in non-dialysis patients; (**4**) Sucra rankings in dialysis patients
- SUCRA rankings confirmed daprodustat and molidustat as top candidates for hepcidin suppression, aligning with pairwise results. vadadustat also scored favorably but with greater uncertainty. ESA and enarodustat ranked lower in probabilistic terms, highlighting the contrast between statistical effect and ranking uncertainty (Fig. 6)(sFigure 9 In Supplementary File).

- The analysis of hepcidin, a central regulator of iron homeostasis, included eight HIF-PHIs and control arms (ESA or placebo), with roxadustat (*n* = 7) and ESA forming the network’s backbone. The treatment network showed sufficient connectivity and no statistically significant inconsistency upon node-splitting analysis(sTable 2 In Supplementary File)(Fig. 3).
- SMDs were used to account for variability in assay methods. daprodustat significantly reduced hepcidin compared to ESA (SMD = −0.56; 95% CI: −0.90 to −0.23), indicating a potentially favorable effect on iron availability. Similarly, vadadustat (SMD = −0.79) and molidustat (SMD = −0.75) demonstrated significant reductions versus placebo. roxadustat did not exhibit a meaningful effect compared to placebo (SMD = 0.01), and differences among other active agents were mostly non-significant, reflecting overlapping confidence intervals or limited power (Fig. 6).

#### Serum iron

**Fig. 7:**
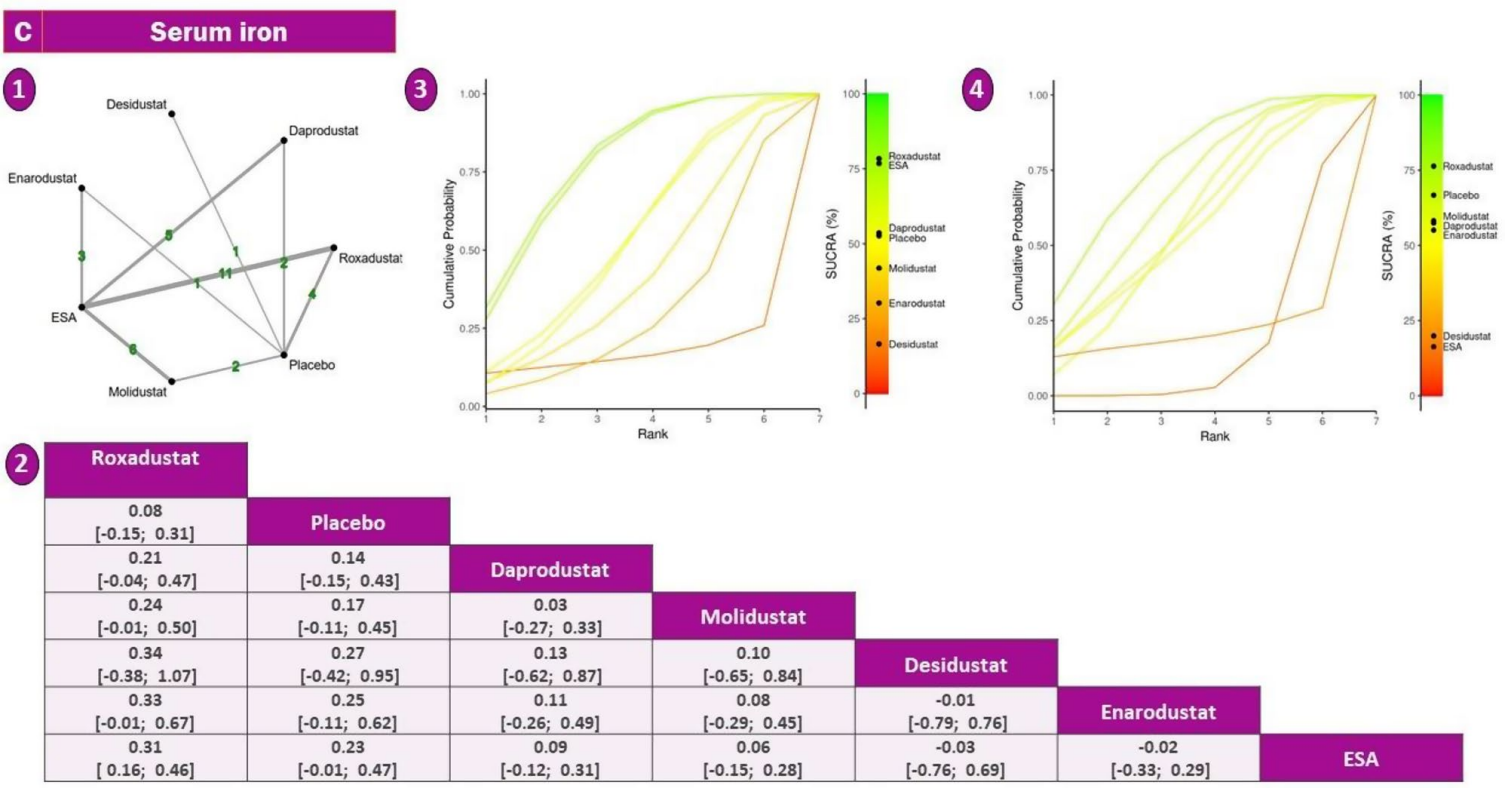
Network geometry, league table (overall), and SUCRA-based ranking of treatments for serum iron. Panels show: (**1**) Network plot of treatment comparisons; (**2**) League table of relative treatment effects; (**3**) Sucra rankings in non-dialysis patients; (**4**) Sucra rankings in dialysis patients
- In SUCRA-based rankings, roxadustat consistently emerged as the most effective agent for increasing serum iron, followed by enarodustat and daprodustat. ESA and placebo ranked lowest, consistent with observed SMDs. Notably, enarodustat’s high SUCRA rank, despite a non-significant effect size, suggests a stable trend across comparisons rather than strong magnitude (sFigure 9 In Supplementary File).

- The serum iron network included seven interventions, with roxadustat (*n* = 11) and ESA as frequent comparators, forming a sufficiently connected network without significant inconsistency (sTable 3 In Supplementary File)(Fig. 3).
- Roxadustat was the only agent that significantly improved serum iron levels compared to ESA (SMD = +0.32; 95% CI: +0.16 to +0.48), indicating a moderate but robust effect. Other agents, including daprodustat and desidustat, demonstrated numerically favorable changes, but none reached statistical significance. ESA itself was associated with a slight numerical reduction (SMD = −0.08), and molidustat and enarodustat produced wide confidence intervals, reflecting limited data and increased uncertainty (Fig. 7).

#### Ferritin

**Fig. 8:**
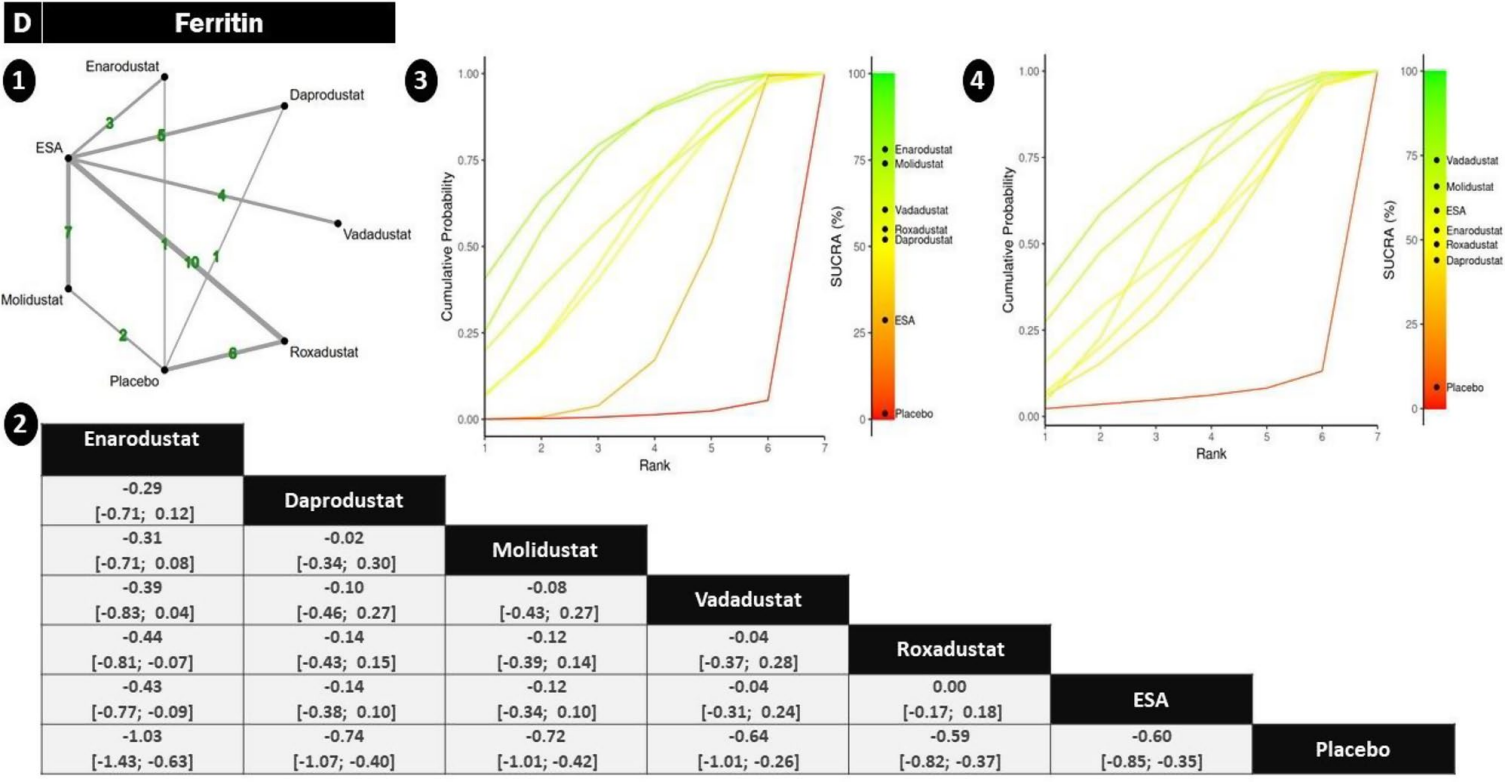
Network geometry, league table (overall), and SUCRA-based ranking of treatments for ferritin. Panels show: (**1**) Network plot of treatment comparisons; (**2**) League table of relative treatment effects; (**3**) Sucra rankings in non-dialysis patients; (**4**) Sucra rankings in dialysis patients

- The ferritin outcome was analyzed across a moderately interconnected network involving roxadustat (*n* = 10), ESA, placebo, and less frequently, enarodustat (*n* = 3) and vadadustat (*n* = 4). No significant inconsistency was identified in the network, supporting the use of consistency models (sTable 4 In Supplementary File)(Fig. 3).
- Among the agents, enarodustat demonstrated the most pronounced numerical reduction in serum ferritin across multiple comparisons, although its confidence intervals often crossed the null due to limited data. daprodustat and molidustat showed consistent downward trends with meaningful reductions. vadadustat and ESA achieved statistically significant reductions compared to placebo. roxadustat also decreased ferritin levels, but with relatively smaller magnitude and wider uncertainty, limiting the robustness of its effect (Fig. 8)

.
- SUCRA rankings reflected these trends, with enarodustat emerging as the top-ranked agent for ferritin lowering, followed by daprodustat and molidustat. vadadustat and ESA occupied intermediate positions, while roxadustat ranked lower, consistent with its modest SMDs, (sFigure 9 In Supplementary File).

#### Total iron binding capacity (TIBC)

**Fig. 9:**
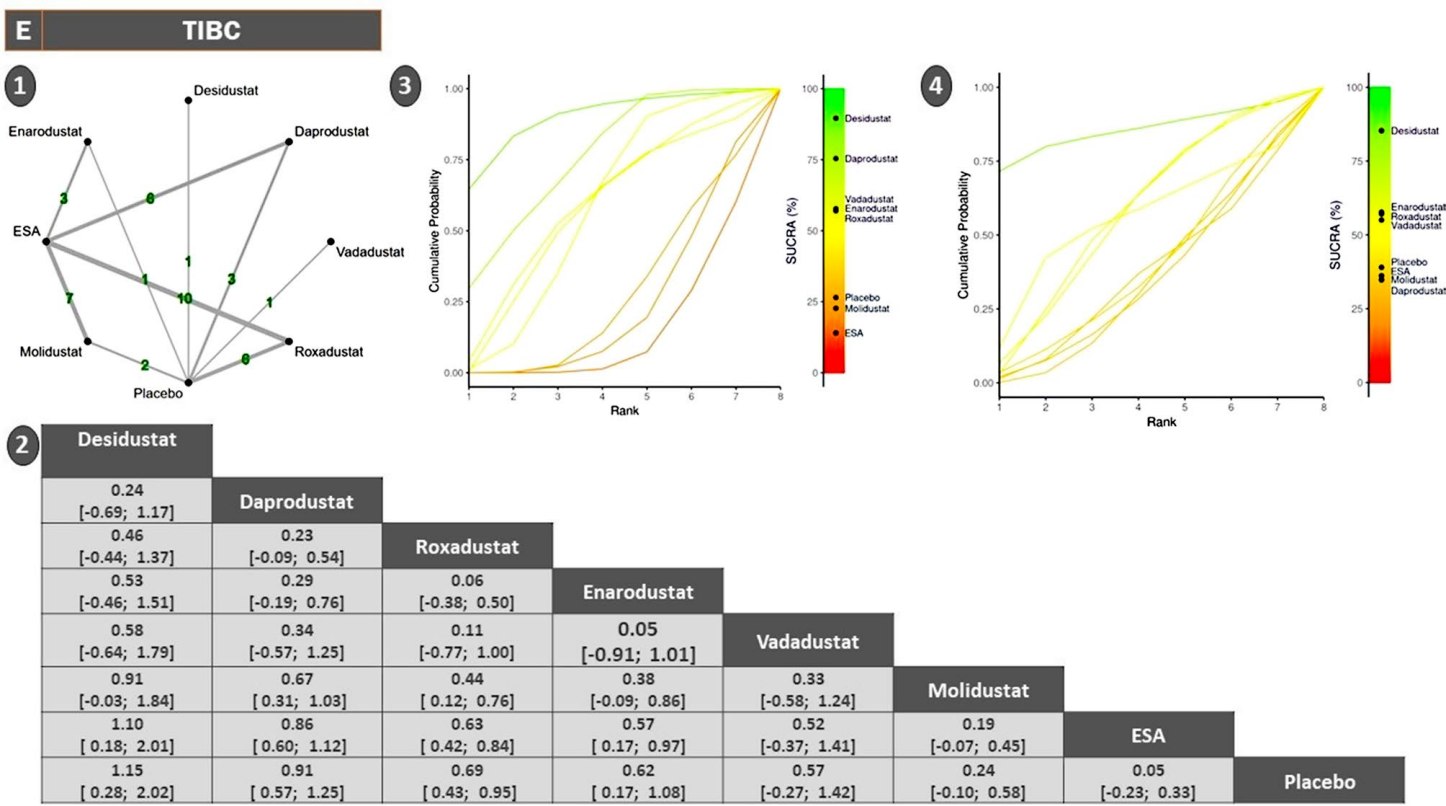
Network geometry, league table (overall), and SUCRA-based ranking of treatments for TIBC. Panels show: (**1**) Network plot of treatment comparisons; (**2**) League table of relative treatment effects, (**3**) Sucra rankings in non-dialysis patients, (**4**) Sucra rankings in dialysis patients

- The TIBC network included seven agents with roxadustat (*n* = 16) and ESA serving as the most frequently studied comparators. The network structure supported both direct and indirect inferences, and no significant inconsistency was detected across treatment contrasts (sTable 5 In Supplementary File)(Fig. 3).
- Among all agents, ESA and molidustat demonstrated the most pronounced effects, with significant increases in TIBC, indicating strong iron mobilization. Daprodustat, roxadustat, and enarodustat showed moderate but statistically non-significant elevations, while desidustat yielded the smallest and least conclusive change, reflecting either reduced biological activity or data limitations (Fig. 9).
- SUCRA rankings presented an interesting inversion: desidustat, daprodustat, and roxadustat ranked highest despite less robust absolute effects. This discrepancy underscores SUCRA’s sensitivity to variance and consistency across network paths rather than effect magnitude alone. ESA and molidustat—despite showing the strongest SMDs—ranked lower, likely due to heterogeneity in indirect estimates or broader confidence intervals in network nodes (sFigure 10 In Supplementary File).

#### Transferrin saturation (tsat)

**Fig. 10:**
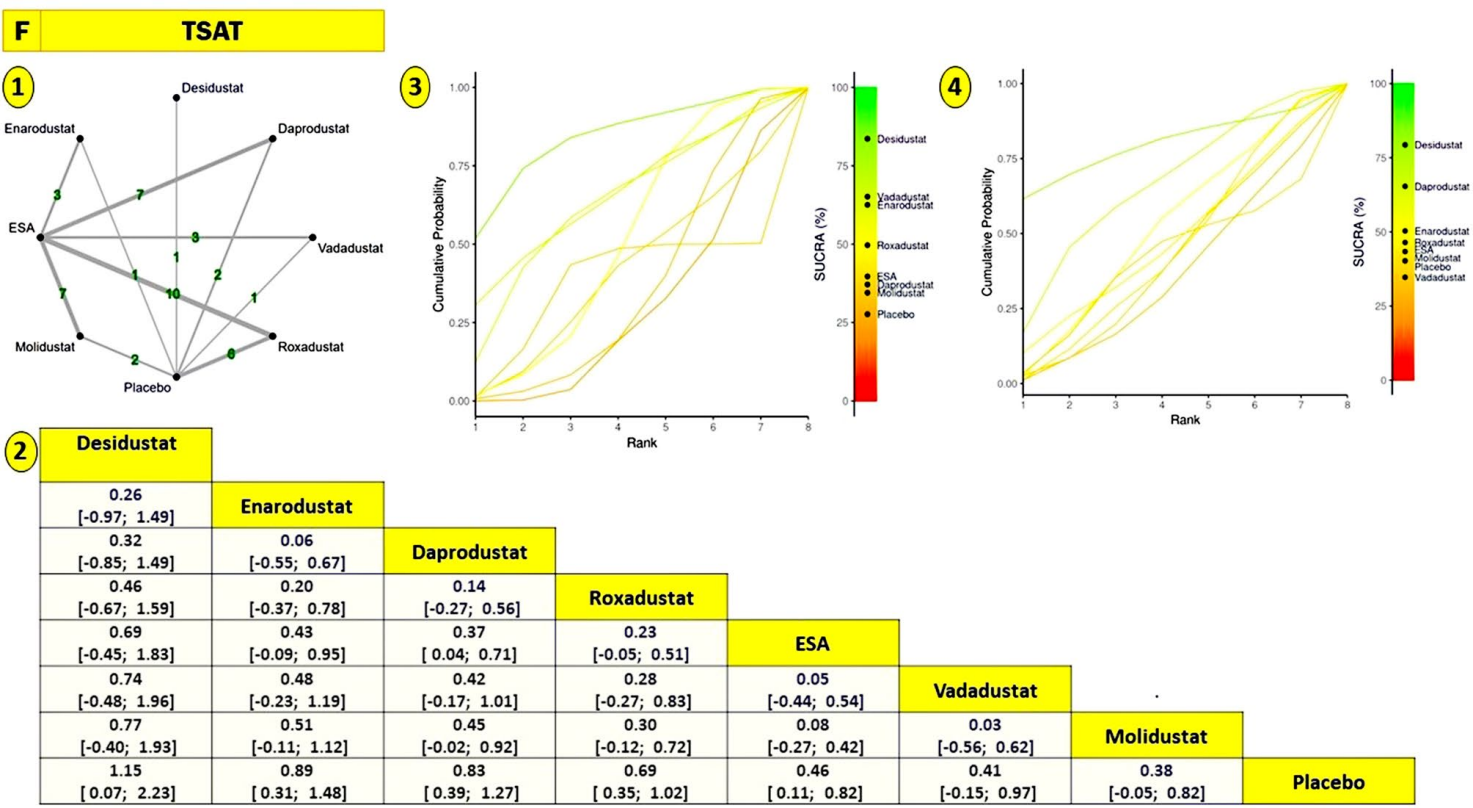
Network geometry, league table (overall), and SUCRA-based ranking of treatments for tsat. Panels show: (**1**) Network plot of treatment comparisons; (**2**) League table of relative treatment effects; (**3**) Sucra rankings in non-dialysis patients; (**4**) Sucra rankings in dialysis patients

- The TSAT network was built with roxadustat (*n* = 16) and ESA anchoring the majority of comparisons. Initial inconsistency assessment indicated discrepancies primarily in trials involving daprodustat versus ESA and placebo. After exclusion of one outlier study, the node-split analysis supported coherence across the network, validating the use of a consistency model for final estimation (sTable 6 In Supplementary File)(Fig. 3).
- Roxadustat and daprodustat both yielded statistically significant improvements in TSAT. roxadustat was notably superior to placebo (SMD = +0.84; 95% CI: +0.43 to +1.25), and daprodustat showed a meaningful effect versus ESA (SMD = +0.37; 95% CI: +0.04 to +0.71). Desidustat recorded the largest numerical benefit (SMD = +1.15), though its wide confidence interval (+0.07 to +2.23) suggested considerable uncertainty. Enarodustat and vadadustat exhibited positive trends, but none reached statistical significance (Fig. 10).
- SUCRA rankings positioned desidustat, enarodustat, and daprodustat at the top, aligning partially with the pairwise outcomes. Roxadustat ranked slightly lower despite strong absolute effects, reflecting SUCRA’s emphasis on consistency and precision rather than effect magnitude alone. Molidustat showed limited efficacy and was ranked near the bottom, reinforcing its weaker profile in iron utilization endpoints (sFigure 10 In Supplementary File).
- Subgroup analysis by dialysis status revealed desidustat as the most stable performer across both NDD and DD populations. Vadadustat and enarodustat ranked favorably in NDD patients, whereas daprodustat’s performance improved markedly among DD patients. (Fig. 10).

#### Safety outcomes and adverse event profiles

- The safety profile of HIF-PH inhibitors was evaluated across a broad range of adverse events, encompassing cardiovascular, metabolic, neurological, gastrointestinal, and fluid-related complications. Network structures were adequately connected in all models, with no major inconsistencies identified through node-splitting (sTable 7 to 20 In Supplementary File) (Fig. 4).
- **Serious cardiovascular events**—including thrombosis, myocardial infarction (MI), stroke, and hypertension—showed limited signals of harm. Most odds ratios (ORs) overlapped the null. Molidustat was the only agent significantly associated with elevated hypertension risk (OR = 2.42; 95% CI: 1.24–4.74). Roxadustat presented numerically higher thrombosis odds than ESA, but this was not statistically significant. Interestingly, Placebo showed significantly higher risk of thrombosis compared to roxadustat (OR = 2.67; 95% CI: 1.14–6.26). SUCRA rankings favored ESA and desidustat for hypertension outcomes, while enarodustat and vadadustat ranked highest for MI, though with high uncertainty (sFigure 1 In Supplementary File) (sFigure 2 In Supplementary File).
- **Vascular and interventional complications** revealed notable trends. Roxadustat significantly increased the odds of vascular occlusion/stenosis compared to ESA (OR = 1.86; 95% CI: 1.11–3.11), flagging a potential safety concern. Red blood cell transfusion need was highest in the Placebo group, with significantly elevated odds versus all active agents. ESA and molidustat consistently ranked as the safest treatments for transfusion avoidance (sFigure 3 In Supplementary File).
- **Electrolyte abnormalities**, specifically hyperkalemia, were infrequent but clinically relevant. Molidustat was the only agent associated with a statistically significant reduction in hyperkalemia risk (OR = 0.37; 95% CI: 0.19–0.73), positioning it favorably among agents with otherwise neutral risk profiles. Most other treatments, including daprodustat and roxadustat, showed slight non-significant elevations in risk. SUCRA rankings reversed the coding to reflect lower incidence as favorable, placing molidustat at the top and desidustat and daprodustat lower (sFigure 4 In Supplementary File).
- **Ophthalmologic and neurologic events** were generally rare and non-significant across trials. For diabetic retinopathy, wide confidence intervals and sparse event data limited interpretability, although molidustat and roxadustat showed numerically higher risks. Headache incidence was higher with daprodustat and vadadustat, though not statistically significant. Desidustat and molidustat showed better tolerability profiles in this domain (sFigure 5 In Supplementary File).
- **Gastrointestinal side effects** demonstrated clearer agent-dependent patterns. Daprodustat was associated with significantly increased risks of both diarrhea (OR = 1.36; 95% CI: 1.17–1.59) and constipation (OR = 1.67; 95% CI: 1.11–2.53), suggesting a less favorable GI profile. Molidustat was uniquely associated with increased nausea (OR = 2.03; 95% CI: 1.15–3.60), while desidustat and vadadustat ranked best for minimizing GI discomfort. Across vomiting and constipation, SUCRA rankings sometimes contradicted effect sizes due to wide CIs and probabilistic modeling (sFigure 6 In Supplementary File) (sFigure 7 In Supplementary File).
- **Edema**, a common fluid-related complication, showed wide but inconclusive estimates across most agents. Roxadustat had the highest numerical odds (OR = 3.04), albeit with broad uncertainty. No agent showed statistically significant differences compared to ESA. SUCRA rankings, adjusted to favor lower risk, placed molidustat and enarodustat at the top, suggesting lower propensity for fluid retention (sFigure 8 In Supplementary File).

#### Assessment of publication bias

Funnel plots were constructed for hemoglobin, serum iron, ferritin, TIBC, TSAT, HDL, and LDL outcomes (sFigures 11 and 12 In Supplementary File). The plots showed overall symmetry, indicating a low risk of publication bias across comparisons versus ESA. Minor asymmetries were noted in some outcomes, such as serum iron and HDL, likely due to small-study effects rather than systematic bias. Overall, the visual inspection supports the reliability of the meta-analysis findings.

## Discussion

### Interpretation of main findings

This comprehensive NMA, encompassing 45 randomized trials and more than 32,000 patients with CKD, elucidates clinically meaningful differences among six HIF-PHIs. While all drugs share a common mechanism—activation of the HIF pathway—they diverge in efficacy and safety profiles. Roxadustat and daprodustat consistently demonstrated superior hemoglobin-raising effects, with daprodustat additionally improving iron indices such as hepcidin, ferritin, and TSAT, particularly in DD patients. These findings underscore that HIF-PHIs should not be treated as a homogeneous class. Importantly, SUCRA-based rankings supported the relative hierarchy of drug efficacy. However, it must be emphasized that SUCRA values represent relative probability, not effect magnitude [65, 66].

### Pharmacologic context, mechanisms, and emerging insights

The heterogeneity in efficacy and safety among HIF-PHIs likely reflects key pharmacological differences rather than class effects. Three mechanistic domains may underlie these variations: isoform selectivity, pharmacokinetics, and iron metabolism modulation. First, differential activation of HIF-1α versus HIF-2α isoforms may result in distinct clinical outcomes. HIF-2α primarily regulates erythropoietin synthesis and iron transporter expression, while HIF-1α influences glycolysis, angiogenesis, and vascular tone [67, 68].

Agents with greater HIF-2α selectivity may achieve more targeted anemia correction with fewer systemic effects, whereas broader HIF-1α activation could explain signals related to vascular or metabolic complications. This may partly account for daprodustat’s stronger impact on hepcidin and TSAT in DD patients, where ESA resistance and inflammation are common [69, 70].

Second, pharmacokinetic properties—including half-life, dosing frequency, and route—also affect clinical performance. Roxadustat, with a relatively long half-life and thrice-weekly administration, may sustain hemoglobin levels more effectively in NDD patients, but prolonged exposure could increase vascular risk in susceptible individuals [26, 28, 71]. Conversely, short-acting agents like molidustat may reduce cumulative exposure and improve safety but require high adherence, especially in outpatient settings [34, 72].

Third, modulation of iron metabolism plays a pivotal role. Agents that suppress hepcidin and improve transferrin saturation can address functional iron deficiency, particularly under inflammatory conditions. Daprodustat and molidustat showed favorable trends in this regard, potentially reducing IV iron need while improving erythropoietic response [14, 56, 57, 67, 68].

Long-term HIF stabilization may also have epigenetic or vascular effects, although this remains speculative and understudied [73, 74]. Importantly, adverse event patterns—such as roxadustat’s vascular occlusion signal or daprodustat’s GI effects—suggest non-equivalent risk profiles. These findings emphasize that HIF-PHIs are not interchangeable.

### Methodological considerations and network limitations

A critical challenge in NMA interpretation is the inherent dependence on indirect comparisons. The lack of true head-to-head RCTs between HIF-PHIs limits the strength of causal inference. Furthermore, the evidence base is disproportionately shaped by large registration trials for roxadustat, daprodustat, and vadadustat, designed for FDA or EMA approval [28, 34, 39, 75]. These trials often employed distinct dosing protocols, comparator choices (placebo vs. ESA), and baseline patient characteristics, introducing heterogeneity into the network. For example, the aggressive dosing of Roxadustat in placebo-controlled trials may partly explain both its high efficacy and elevated thrombotic risk.

To maintain analytical integrity, parameters such as VEGF and lipid profiles—absent in many studies—were excluded from our final synthesis. This aligns with recommendations from regulatory agencies, including the FDA, which emphasize cautious interpretation of NMA results lacking uniform data across arms.

### Safety profiles: clinical and regulatory considerations

This analysis revealed no uniform cardiovascular or neurologic toxicity signals across the HIF-PHI class. Nonetheless, specific agents presented concerns. Although roxadustat was not approved by the FDA for NDD patients due to initial safety concerns, subsequent trials have provided additional safety data. Regulatory decisions should be interpreted alongside evolving evidence. Vadadustat also failed to gain approval in the U.S. and Europe for similar reasons. Notably, these safety signals were not uniformly detected in pooled analyses, reflecting potential differences in event adjudication, trial duration, and population risk profiles.

Molidustat stood out for its protective signal against hyperkalemia. The underlying mechanism remains speculative but may involve reduced ESA or IV iron exposure, or modulation of renal potassium channels. On the contrary, mild elevations in potassium levels with roxadustat and daprodustat may result from HIF-induced changes in renal ROMK and BK channel expression, particularly under concurrent RAAS blockade [76].

The need for transfusion—used as a surrogate marker of therapeutic inadequacy—was paradoxically higher in some roxadustat studies. This likely reflects methodological artifacts: placebo-arm patients were often switched to ESA upon dialysis initiation, while roxadustat patients remained on treatment despite ongoing blood loss and access-related complications.

Adherence is another important yet underexplored variable. Roxadustat’s thrice-weekly regimen, often administered in dialysis units, may enhance compliance. In contrast, daprodustat’s once-daily oral dosing—though potentially more physiologic—requires patient-level adherence, which may be affected by polypharmacy or cognitive burden [10].

Finally, rare long-term safety outcomes such as malignancy or retinopathy were insufficiently reported across trials. Early real-world data suggest no major divergence from trial findings, but continued pharmacovigilance is critical.

### Comparison with prior literature and real-world data

Our NMA expands upon previous studies by offering a more detailed comparison among six HIF-PHIs across over 32,000 CKD patients. Unlike Ren et al. (2024) [15], which found similar efficacy among HIF-PHIs, we identified roxadustat as the most effective for hemoglobin, particularly in non-dialysis patients, while daprodustat showed higher efficacy in dialysis-dependent populations. For hepcidin, our findings align with Yang et al. (2023) [70], confirming daprodustat and molidustat as top suppressors. However, Chen et al. (2023) [77] ranked roxadustat higher, possibly due to dialysis-only data. In terms of iron indices, our network showed agent-specific variability in ferritin, TIBC, and TSAT, not always consistent with SUCRA rankings. Safety results matched prior NMAs, showing no consistent increase in cardiovascular risk, though roxadustat showed a higher rate of vascular complications. Variations across networks likely reflect differences in populations, endpoints, and inconsistency handling, but all support the effectiveness of HIF-PHIs with distinct profiles.

Notably, emerging real-world evidence (RWE) from Japan and Europe suggests that adherence, adverse event patterns, and response durability may differ from RCT findings. Integration of RWE with NMA can bridge the gap between efficacy and effectiveness, and should be prioritized in future comparative studies.

### Strengths, limitations, and future directions

This NMA benefits from its comprehensive scope, inclusion of over 32,000 patients from 45 RCTs, and application of both frequentist and Bayesian statistical frameworks. The prespecified protocol, stratification by dialysis status, and incorporation of clinically meaningful biomarkers such as hepcidin, ferritin, and TSAT enhance its translational value.

Nevertheless, several limitations should be acknowledged. First, between-trial heterogeneity in baseline inflammation, ESA regimens, iron status and route of administration, dialysis vintage, and geographic practice patterns introduces potential confounding that cannot be fully addressed in study-level synthesis. Second, the lack of individual patient data (IPD) limits subgroup analyses beyond dialysis dependence and prevents adjustment for key modifiers such as C-reactive protein, RAAS inhibitor use, or baseline iron stores. Third, the use of SMDs for iron biomarkers—though methodologically appropriate given assay variability—reduces clinical interpretability. Additionally, imputation of missing standard deviations based on assumed pre–post correlations introduces statistical uncertainty. Network geometry was unbalanced, with a concentration of evidence around roxadustat, daprodustat, and vadadustat, while data for molidustat, enarodustat, and desidustat were sparse, limiting precision and inflating SUCRA variability. Moreover, safety endpoints were inconsistently reported, often underpowered, and subject to bias in open-label trials. Rare events such as myocardial infarction, stroke, malignancy, and retinopathy remain insufficiently characterized.

Future studies should prioritize direct head-to-head comparisons among HIF-PHIs, individual patient-level analyses to explore effect modification, and longer-term follow-up for vascular and metabolic outcomes. Integration of real-world evidence and biomarker-guided treatment strategies—including IL-6 levels, HIF isoform activity, and genetic variants of EPO or iron regulators—may enable personalized therapy. While oral agents may improve anemia care in low-resource settings, current evidence is insufficient to conclude that any specific HIF-PHI, including desidustat, offers superior long-term safety over others.

### Conclusion

In summary, HIF-PHIs represent a promising yet heterogeneous class of therapies for CKD-related anemia. Roxadustat and Daprodustat emerge as the most effective agents, with Daprodustat offering additional advantages in iron mobilization. However, safety concerns—especially cardiovascular and metabolic—must inform therapeutic choices. Personalized selection based on dialysis status, inflammation burden, access considerations, and adherence potential is critical. Future research should move beyond hemoglobin endpoints toward mechanistic clarity, biomarker-based stratification, and global implementation strategies to optimize care.

## Electronic supplementary material

Below is the link to the electronic supplementary material.

Supplementary Material 1

Supplementary Material 2

## Data Availability

All data generated or analyzed during this study are included in this published article and its supplementary information files.

## Abbreviations

CKD: Chronic kidney disease
Hb: Hemoglobin
ESA: Erythropoiesis-stimulating agent
HIF-PHI: Hypoxia-inducible factor prolyl hydroxylase inhibitor
RCT: Randomized controlled trial
NDD: Non-dialysis dependent
DD: Dialysis-dependent
TIBC: Total iron-binding capacity
TSAT: Transferrin saturation
VEGF: Vascular endothelial growth factor
SUCRA: Surface under the cumulative ranking curve
OR: Odds ratio
CI: Confidence interval
SMD: Standardized mean difference
