# Supplementary material for: Network meta-analysis of HIF-prolyl hydroxylase inhibitors for anemia in dialysis-dependent and non-dialysis CKD: effects on hemoglobin, iron markers, and adverse clinical outcomes
Source: BMC Nephrol. 2025 Nov 14;26:638. doi: 10.1186/s12882-025-04561-x (PMC12619315; doi:10.1186/s12882-025-04561-x)
Supplement: Supplementary file 1 — Supplementary Material 1 [file 12882_2025_4561_MOESM1_ESM.docx]

|  | Systematic search strategy for different databases |
| --- | --- |
| Pubmed | **#1. Intervention (HIF-PHIs):**  GSK1278863[Supplementary Concept]  OR roxadustat[Supplementary Concept]  OR vadadustat[Supplementary Concept]  OR molidustat[Supplementary Concept]  OR enarodustat[Supplementary Concept]  OR HIF-PHI[tiab]  OR HIF stabilizer*[tiab]  OR HIF prolyl hydroxylase inhibitor*[tiab]  OR daprodustat[tiab]  OR roxadustat[tiab]  OR vadadustat[tiab]  OR molidustat[tiab]  OR enarodustat[tiab]  **#2. Outcomes (Biomarkers + Adverse Events):**  hemoglobin[tiab]  OR ferritin[tiab] OR hepcidin[tiab] OR iron[tiab] OR serum iron[tiab] OR TIBC[tiab] OR TSAT[tiab] OR VEGF[tiab] OR HDL[tiab] OR LDL[tiab] OR cholesterol[tiab] OR adverse event*[tiab] OR cancer[tiab**]** OR neoplasm*[tiab] OR edema[tiab] OR thrombosis[tiab] OR fatigue[tiab] OR stroke[tiab] OR myocardial infarction[tiab] OR heart failure[tiab] OR retinopathy[tiab] OR headache[tiab] OR nausea[tiab] OR vomiting[tiab] OR diarrhea[tiab] OR constipation[tiab] OR transfusion[tiab] OR Anemia[Mesh]  **#3. Population (CKD / ESRD / dialysis):**  Kidney Diseases[Mesh] OR Renal Insufficiency, Chronic[Mesh] OR chronic kidney disease[tiab] OR CKD[tiab] OR ESRD[tiab] OR end-stage renal disease[tiab] OR dialysis[tiab] OR renal failure[tiab]  **#4. Study design filter (RCT):**  randomized controlled trial[pt] OR randomized[tiab] OR placebo[tiab] OR RCT[tiab]  **#5. Final combined search:**  #1 AND #2 AND #3 AND #4 |
| EMBASE | #1 'daprodustat'/exp OR 'roxadustat'/exp OR 'vadadustat'/exp OR 'molidustat'/exp OR 'enarodustat'/exp  OR 'HIF stabilizer*':ti,ab OR 'HIF-PHI':ti,ab OR 'HIF prolyl hydroxylase inhibitor*':ti,ab  #2 hemoglobin:ti,ab OR ferritin:ti,ab OR hepcidin:ti,ab OR 'serum iron':ti,ab OR TIBC:ti,ab OR TSAT:ti,ab  OR VEGF:ti,ab OR HDL:ti,ab OR LDL:ti,ab OR cholesterol:ti,ab  OR 'adverse event*':ti,ab OR cancer:ti,ab OR neoplasm*:ti,ab  OR edema:ti,ab OR thrombosis:ti,ab OR fatigue:ti,ab OR stroke:ti,ab  OR 'myocardial infarction':ti,ab OR 'heart failure':ti,ab OR retinopathy:ti,ab  OR headache:ti,ab OR nausea:ti,ab OR vomiting:ti,ab OR diarrhea:ti,ab  OR constipation:ti,ab OR transfusion:ti,ab  #3 'chronic kidney disease'/exp OR 'renal insufficiency'/exp OR 'dialysis'/exp  OR 'chronic kidney disease':ti,ab OR CKD:ti,ab OR ESRD:ti,ab  #4 randomized controlled trial/exp OR randomized:ti,ab OR placebo:ti,ab OR RCT:ti,ab  #5 #1 AND #2 AND #3 AND #4 |
| Scopus | #1 TITLE-ABS-KEY(daprodustat OR roxadustat OR vadadustat OR molidustat OR enarodustat  OR HIF-PHI OR HIF stabilizer* OR HIF prolyl hydroxylase inhibitor*)  #2 TITLE-ABS-KEY(hemoglobin OR ferritin OR hepcidin OR serum iron OR TIBC OR TSAT OR VEGF  OR HDL OR LDL OR cholesterol OR adverse event* OR cancer OR neoplasm* OR edema  OR thrombosis OR fatigue OR stroke OR myocardial infarction OR heart failure  OR retinopathy OR headache OR nausea OR vomiting OR diarrhea OR constipation OR transfusion)  #3 TITLE-ABS-KEY(chronic kidney disease OR CKD OR ESRD OR end-stage renal disease OR dialysis OR renal failure)  #4 TITLE-ABS-KEY(randomized OR placebo OR controlled trial OR RCT)  #5 #1 AND #2 AND #3 AND #4 |
| Web of Science | #1 TS=(daprodustat OR roxadustat OR vadadustat OR molidustat OR enarodustat  OR HIF-PHI OR HIF stabilizer* OR HIF prolyl hydroxylase inhibitor*)  #2 TS=(hemoglobin OR ferritin OR hepcidin OR serum iron OR TIBC OR TSAT OR VEGF  OR HDL OR LDL OR cholesterol OR adverse event* OR cancer OR neoplasm* OR edema  OR thrombosis OR fatigue OR stroke OR myocardial infarction OR heart failure  OR retinopathy OR headache OR nausea OR vomiting OR diarrhea OR constipation OR transfusion)  #3 TS=(chronic kidney disease OR CKD OR ESRD OR end-stage renal disease OR dialysis OR renal failure)  #4 TS=(randomized OR placebo OR controlled trial OR RCT)  #5 #1 AND #2 AND #3 AND #4 |
| Cochrane Library | #1 daprodustat OR roxadustat OR vadadustat OR molidustat OR enarodustat  OR HIF stabilizer* OR HIF-PHI OR HIF prolyl hydroxylase inhibitor*  #2 hemoglobin OR ferritin OR hepcidin OR serum iron OR TIBC OR TSAT OR VEGF  OR HDL OR LDL OR cholesterol OR adverse event* OR cancer OR neoplasm* OR edema  OR thrombosis OR fatigue OR stroke OR myocardial infarction OR heart failure  OR retinopathy OR headache OR nausea OR vomiting OR diarrhea OR constipation OR transfusion  #3 chronic kidney disease OR CKD OR ESRD OR end-stage renal disease OR dialysis OR renal failure  #4 #1 AND #2 AND #3 |
| ClinicalKey | #1 daprodustat OR roxadustat OR vadadustat OR molidustat OR enarodustat  OR HIF stabilizer* OR HIF-PHI OR HIF prolyl hydroxylase inhibitor*  #2 hemoglobin OR ferritin OR hepcidin OR serum iron OR TIBC OR TSAT OR VEGF  OR HDL OR LDL OR cholesterol OR adverse event* OR cancer OR neoplasm* OR edema  OR thrombosis OR fatigue OR stroke OR myocardial infarction OR heart failure  OR retinopathy OR headache OR nausea OR vomiting OR diarrhea OR constipation OR transfusion  #3 chronic kidney disease OR CKD OR ESRD OR end-stage renal disease OR dialysis OR renal failure  #4 randomized OR placebo OR controlled trial OR RCT  #5 #1 AND #2 AND #3 AND #4 |
| ScienceDirect | #1 daprodustat OR roxadustat OR vadadustat OR molidustat OR enarodustat  OR HIF stabilizer* OR HIF-PHI OR HIF prolyl hydroxylase inhibitor*  #2 hemoglobin OR ferritin OR hepcidin OR serum iron OR TIBC OR TSAT OR VEGF  OR HDL OR LDL OR cholesterol OR adverse event* OR cancer OR neoplasm* OR edema  OR thrombosis OR fatigue OR stroke OR myocardial infarction OR heart failure  OR retinopathy OR headache OR nausea OR vomiting OR diarrhea OR constipation OR transfusion  #3 chronic kidney disease OR CKD OR ESRD OR end-stage renal disease OR dialysis OR renal failure  #4 randomized OR placebo OR controlled trial OR RCT  #5 #1 AND #2 AND #3 AND #4 |
| Google Scholar | #1 HIF-PHI OR HIF stabilizer OR daprodustat OR roxadustat OR vadadustat OR molidustat OR enarodustat  #2 hemoglobin OR ferritin OR hepcidin OR VEGF OR adverse event OR thrombosis OR cancer  #3 chronic kidney disease OR CKD OR dialysis OR ESRD  #4 randomized OR RCT OR placebo  #5 #1 AND #2 AND #3 AND #4 |
| Google | daprodustat roxadustat vadadustat molidustat enarodustat HIF-PHI CKD dialysis anemia trial |
