# Supplementary material for: Network meta-analysis of HIF-prolyl hydroxylase inhibitors for anemia in dialysis-dependent and non-dialysis CKD: effects on hemoglobin, iron markers, and adverse clinical outcomes
Source: BMC Nephrol. 2025 Nov 14;26:638. doi: 10.1186/s12882-025-04561-x (PMC12619315; doi:10.1186/s12882-025-04561-x)
Supplement: Supplementary file 2 — Supplementary Material 2 [file 12882_2025_4561_MOESM2_ESM.docx]

| **sTable 1.** Assessment of inconsistency in the network meta-analysis for hemoglobin (Hb) | | | | | | | | |
| --- | --- | --- | --- | --- | --- | --- | --- | --- |
| Comparison | No.Studies | NMA | Direct | Indirect | Difference | Diff_95CI_lower | Diff_95CI_upper | pValue |
| Daprodustat:Desidustat | 0 | 0/002 |  | 0/002 |  |  |  |  |
| Daprodustat:Enarodustat | 0 | 0/280 |  | 0/280 |  |  |  |  |
| Daprodustat:ESA | 7 | 0/170 | 0/189 | 0/095 | 0/093 | -0/692 | 0/879 | 0/816 |
| Daprodustat:Molidustat | 0 | 0/211 |  | 0/211 |  |  |  |  |
| Daprodustat:Placebo | 3 | 1/662 | 1/607 | 1/700 | -0/093 | -0/879 | 0/692 | 0/816 |
| Daprodustat:Roxadustat | 0 | -0/177 |  | -0/177 |  |  |  |  |
| Daprodustat:Vadadustat | 0 | 0/108 |  | 0/108 |  |  |  |  |
| Desidustat:Enarodustat | 0 | 0/278 |  | 0/278 |  |  |  |  |
| Desidustat:ESA | 2 | 0/167 | 0/140 | 0/304 | -0/165 | -1/810 | 1/481 | 0/845 |
| Desidustat:Molidustat | 0 | 0/209 |  | 0/209 |  |  |  |  |
| Desidustat:Placebo | 1 | 1/659 | 1/790 | 1/625 | 0/165 | -1/481 | 1/810 | 0/845 |
| Desidustat:Roxadustat | 0 | -0/179 |  | -0/179 |  |  |  |  |
| Desidustat:Vadadustat | 0 | 0/106 |  | 0/106 |  |  |  |  |
| Enarodustat:ESA | 3 | -0/111 | -0/334 | 0/690 | -1/024 | -2/199 | 0/151 | 0/088 |
| Enarodustat:Molidustat | 0 | -0/069 |  | -0/069 |  |  |  |  |
| Enarodustat:Placebo | 1 | 1/381 | 2/100 | 1/076 | 1/024 | -0/151 | 2/199 | 0/088 |
| Enarodustat:Roxadustat | 0 | -0/457 |  | -0/457 |  |  |  |  |
| Enarodustat:Vadadustat | 0 | -0/172 |  | -0/172 |  |  |  |  |
| Molidustat:ESA | 9 | -0/041 | -0/035 | -0/103 | 0/068 | -1/014 | 1/150 | 0/902 |
| Placebo:ESA | 0 | -1/492 |  | -1/492 |  |  |  |  |
| Roxadustat:ESA | 9 | 0/347 | 0/383 | 0/235 | 0/148 | -0/497 | 0/793 | 0/653 |
| Vadadustat:ESA | 5 | 0/062 | 0/103 | -0/085 | 0/187 | -0/701 | 1/076 | 0/680 |
| Molidustat:Placebo | 2 | 1/451 | 1/395 | 1/463 | -0/068 | -1/150 | 1/014 | 0/902 |
| Molidustat:Roxadustat | 0 | -0/388 |  | -0/388 |  |  |  |  |
| Molidustat:Vadadustat | 0 | -0/103 |  | -0/103 |  |  |  |  |
| Placebo:Roxadustat | 7 | -1/839 | -1/790 | -1/938 | 0/148 | -0/497 | 0/793 | 0/653 |
| Placebo:Vadadustat | 2 | -1/554 | -1/436 | -1/623 | 0/187 | -0/701 | 1/076 | 0/680 |
| Roxadustat:Vadadustat | 0 | 0/285 |  | 0/285 |  |  |  |  |

| **sTable 2** Assessment of inconsistency in the network meta-analysis for Hepcidin | | | | | | | |  |
| --- | --- | --- | --- | --- | --- | --- | --- | --- |
| Comparison | No.Studies | NMA | Direct | Indirect | Difference | Diff_95CI_lower | Diff_95CI_upper | pValue |
| Daprodustat:Desidustat | 0 | -0/441 |  | -0/441 |  |  |  |  |
| Daprodustat:Enarodustat | 0 | -0/538 |  | -0/538 |  |  |  |  |
| Daprodustat:ESA | 4 | -0/582 | -0/564 | -0/735 | 0/171 | -0/849 | 1/190 | 0/743 |
| Daprodustat:Molidustat | 0 | -0/250 |  | -0/250 |  |  |  |  |
| Daprodustat:Placebo | 1 | -0/708 | -0/843 | -0/672 | -0/171 | -1/190 | 0/849 | 0/743 |
| Daprodustat:Roxadustat | 0 | -0/460 |  | -0/460 |  |  |  |  |
| Daprodustat:Vadadustat | 0 | -0/297 |  | -0/297 |  |  |  |  |
| Desidustat:Enarodustat | 0 | -0/097 |  | -0/097 |  |  |  |  |
| Desidustat:ESA | 1 | -0/141 | -0/099 | -0/214 | 0/114 | -0/977 | 1/206 | 0/837 |
| Desidustat:Molidustat | 0 | 0/191 |  | 0/191 |  |  |  |  |
| Desidustat:Placebo | 1 | -0/267 | -0/329 | -0/215 | -0/114 | -1/206 | 0/977 | 0/837 |
| Desidustat:Roxadustat | 0 | -0/019 |  | -0/019 |  |  |  |  |
| Desidustat:Vadadustat | 0 | 0/144 |  | 0/144 |  |  |  |  |
| Enarodustat:ESA | 1 | -0/044 | -0/044 |  |  |  |  |  |
| Enarodustat:Molidustat | 0 | 0/288 |  | 0/288 |  |  |  |  |
| Enarodustat:Placebo | 0 | -0/169 |  | -0/169 |  |  |  |  |
| Enarodustat:Roxadustat | 0 | 0/078 |  | 0/078 |  |  |  |  |
| Enarodustat:Vadadustat | 0 | 0/242 |  | 0/242 |  |  |  |  |
| Molidustat:ESA | 3 | -0/332 | -0/181 | -0/728 | 0/547 | -0/227 | 1/321 | 0/166 |
| Placebo:ESA | 0 | 0/126 |  | 0/126 |  |  |  |  |
| Roxadustat:ESA | 7 | -0/122 | -0/261 | 0/493 | -0/754 | -1/372 | -0/135 | 0/017 |
| Vadadustat:ESA | 2 | -0/285 | -0/120 | -0/756 | 0/636 | -0/256 | 1/529 | 0/162 |
| Molidustat:Placebo | 2 | -0/457 | -0/746 | -0/199 | -0/547 | -1/321 | 0/227 | 0/166 |
| Molidustat:Roxadustat | 0 | -0/210 |  | -0/210 |  |  |  |  |
| Molidustat:Vadadustat | 0 | -0/047 |  | -0/047 |  |  |  |  |
| Placebo:Roxadustat | 4 | 0/248 | -0/010 | 0/743 | -0/754 | -1/372 | -0/135 | 0/017 |
| Placebo:Vadadustat | 1 | 0/411 | 0/793 | 0/157 | 0/636 | -0/256 | 1/529 | 0/162 |
| Roxadustat:Vadadustat | 0 | 0/163 |  | 0/163 |  |  |  |  |

| **sTable 3.** Assessment of inconsistency in the network meta-analysis for Serum Iron | | | | | | | | |
| --- | --- | --- | --- | --- | --- | --- | --- | --- |
| Comparison | No.Studis | NMA | Direct | Indirect | Difference | Diff_95CI_lower | Diff_95CI_upper | pValue |
| Daprodustat:Desidustat | 0 | 0/128 |  | 0/128 |  |  |  |  |
| Daprodustat:Enarodustat | 0 | 0/114 |  | 0/114 |  |  |  |  |
| Daprodustat:ESA | 5 | 0/093 | 0/132 | -0/168 | 0/300 | -0/332 | 0/932 | 0/352 |
| Daprodustat:Molidustat | 0 | 0/030 |  | 0/030 |  |  |  |  |
| Daprodustat:Placebo | 2 | -0/139 | -0/348 | -0/048 | -0/300 | -0/932 | 0/332 | 0/352 |
| Daprodustat:Roxadustat | 0 | -0/215 |  | -0/215 |  |  |  |  |
| Desidustat:Enarodustat | 0 | -0/014 |  | -0/014 |  |  |  |  |
| Desidustat:ESA | 0 | -0/035 |  | -0/035 |  |  |  |  |
| Desidustat:Molidustat | 0 | -0/098 |  | -0/098 |  |  |  |  |
| Desidustat:Placebo | 1 | -0/267 | -0/267 |  |  |  |  |  |
| Desidustat:Roxadustat | 0 | -0/343 |  | -0/343 |  |  |  |  |
| Enarodustat:ESA | 3 | -0/021 | -0/079 | 0/301 | -0/379 | -1/240 | 0/481 | 0/388 |
| Enarodustat:Molidustat | 0 | -0/084 |  | -0/084 |  |  |  |  |
| Enarodustat:Placebo | 1 | -0/253 | 0/037 | -0/343 | 0/379 | -0/481 | 1/240 | 0/388 |
| Enarodustat:Roxadustat | 0 | -0/329 |  | -0/329 |  |  |  |  |
| Molidustat:ESA | 6 | 0/063 | 0/024 | 0/271 | -0/247 | -0/836 | 0/341 | 0/410 |
| Placebo:ESA | 0 | 0/232 |  | 0/232 |  |  |  |  |
| Roxadustat:ESA | 11 | 0/308 | 0/320 | 0/211 | 0/109 | -0/370 | 0/588 | 0/656 |
| Molidustat:Placebo | 2 | -0/169 | -0/012 | -0/259 | 0/247 | -0/341 | 0/836 | 0/410 |
| Molidustat:Roxadustat | 0 | -0/245 |  | -0/245 |  |  |  |  |
| Placebo:Roxadustat | 4 | -0/076 | -0/037 | -0/146 | 0/109 | -0/370 | 0/588 | 0/656 |

| **sTable 4.** Assessment of inconsistency in the network meta-analysis for Ferritin | | | | | | | | |
| --- | --- | --- | --- | --- | --- | --- | --- | --- |
| Comparison | No.Studis | NMA | Direct | Indirect | Difference | Diff_95CI_lower | Diff_95CI_upper | pValue |
| Daprodustat:Enarodustat | 0 | 0/295 |  | 0/295 |  |  |  |  |
| Daprodustat:ESA | 5 | -0/136 | -0/138 | -0/119 | -0/019 | -0/924 | 0/885 | 0/967 |
| Daprodustat:Molidustat | 0 | -0/018 |  | -0/018 |  |  |  |  |
| Daprodustat:Placebo | 1 | -0/735 | -0/719 | -0/738 | 0/019 | -0/885 | 0/924 | 0/967 |
| Daprodustat:Roxadustat | 0 | -0/141 |  | -0/141 |  |  |  |  |
| Daprodustat:Vadadustat | 0 | -0/099 |  | -0/099 |  |  |  |  |
| Enarodustat:ESA | 3 | -0/431 | -0/325 | -1/082 | 0/757 | -0/214 | 1/729 | 0/127 |
| Enarodustat:Molidustat | 0 | -0/313 |  | -0/313 |  |  |  |  |
| Enarodustat:Placebo | 1 | -1/030 | -1/627 | -0/869 | -0/757 | -1/729 | 0/214 | 0/127 |
| Enarodustat:Roxadustat | 0 | -0/436 |  | -0/436 |  |  |  |  |
| Enarodustat:Vadadustat | 0 | -0/394 |  | -0/394 |  |  |  |  |
| ESA:Molidustat | 7 | 0/118 | 0/089 | 0/301 | -0/212 | -0/836 | 0/412 | 0/506 |
| ESA:Placebo | 0 | -0/599 |  | -0/599 |  |  |  |  |
| ESA:Roxadustat | 10 | -0/005 | 0/040 | -0/322 | 0/362 | -0/161 | 0/886 | 0/175 |
| ESA:Vadadustat | 4 | 0/037 | 0/037 |  |  |  |  |  |
| Molidustat:Placebo | 2 | -0/717 | -0/857 | -0/645 | -0/212 | -0/836 | 0/412 | 0/506 |
| Molidustat:Roxadustat | 0 | -0/123 |  | -0/123 |  |  |  |  |
| Molidustat:Vadadustat | 0 | -0/081 |  | -0/081 |  |  |  |  |
| Roxadustat:Placebo | 6 | -0/594 | -0/509 | -0/871 | 0/362 | -0/161 | 0/886 | 0/175 |
| Vadadustat:Placebo | 0 | -0/636 |  | -0/636 |  |  |  |  |
| Roxadustat:Vadadustat | 0 | 0/042 |  | 0/042 |  |  |  |  |

| **sTable 5.** Assessment of inconsistency in the network meta-analysis for TIBC | | | | | | | | |
| --- | --- | --- | --- | --- | --- | --- | --- | --- |
| Comparison | No.Studis | NMA | Direct | Indirect | Difference | Diff_95CI_lower | Diff_95CI_upper | pValue |
| Daprodustat:Desidustat | 0 | -0/239 |  | -0/239 |  |  |  |  |
| Daprodustat:Enarodustat | 0 | 0/287 |  | 0/287 |  |  |  |  |
| Daprodustat:ESA | 6 | 0/859 | 0/914 | 0/599 | 0/315 | -0/378 | 1/007 | 0/373 |
| Daprodustat:Molidustat | 0 | 0/671 |  | 0/671 |  |  |  |  |
| Daprodustat:Placebo | 3 | 0/911 | 0/718 | 1/032 | -0/315 | -1/007 | 0/378 | 0/373 |
| Daprodustat:Roxadustat | 0 | 0/226 |  | 0/226 |  |  |  |  |
| Daprodustat:Vadadustat | 0 | 0/339 |  | 0/339 |  |  |  |  |
| Desidustat:Enarodustat | 0 | 0/526 |  | 0/526 |  |  |  |  |
| Desidustat:ESA | 0 | 1/098 |  | 1/098 |  |  |  |  |
| Desidustat:Molidustat | 0 | 0/910 |  | 0/910 |  |  |  |  |
| Desidustat:Placebo | 1 | 1/150 | 1/150 |  |  |  |  |  |
| Desidustat:Roxadustat | 0 | 0/465 |  | 0/465 |  |  |  |  |
| Desidustat:Vadadustat | 0 | 0/578 |  | 0/578 |  |  |  |  |
| Enarodustat:ESA | 3 | 0/571 | 0/825 | -0/595 | 1/419 | 0/370 | 2/469 | 0/008 |
| Enarodustat:Molidustat | 0 | 0/383 |  | 0/383 |  |  |  |  |
| Enarodustat:Placebo | 1 | 0/624 | -0/431 | 0/988 | -1/419 | -2/469 | -0/370 | 0/008 |
| Enarodustat:Roxadustat | 0 | -0/061 |  | -0/061 |  |  |  |  |
| Enarodustat:Vadadustat | 0 | 0/052 |  | 0/052 |  |  |  |  |
| Molidustat:ESA | 7 | 0/188 | 0/142 | 0/437 | -0/295 | -1/017 | 0/427 | 0/423 |
| Placebo:ESA | 0 | -0/053 |  | -0/053 |  |  |  |  |
| Roxadustat:ESA | 10 | 0/633 | 0/559 | 0/998 | -0/439 | -1/003 | 0/125 | 0/127 |
| Vadadustat:ESA | 0 | 0/520 |  | 0/520 |  |  |  |  |
| Molidustat:Placebo | 2 | 0/241 | 0/435 | 0/140 | 0/295 | -0/427 | 1/017 | 0/423 |
| Molidustat:Roxadustat | 0 | -0/445 |  | -0/445 |  |  |  |  |
| Molidustat:Vadadustat | 0 | -0/332 |  | -0/332 |  |  |  |  |
| Placebo:Roxadustat | 6 | -0/685 | -0/820 | -0/381 | -0/439 | -1/003 | 0/125 | 0/127 |
| Placebo:Vadadustat | 1 | -0/572 | -0/572 |  |  |  |  |  |
| Roxadustat:Vadadustat | 0 | 0/113 |  | 0/113 |  |  |  |  |

| **sTable 6.** Assessment of inconsistency in the network meta-analysis for TSAT | | | | | | | | | |
| --- | --- | --- | --- | --- | --- | --- | --- | --- | --- |
| Comparison | No.Studis | NMA | Direct | Indirect | Difference | Diff_95CI_lower | Diff_95CI_upper | pValue |  |
| Daprodustat:Desidustat | 0 | -0/317 |  | -0/317 |  |  |  |  |  |
| Daprodustat:Enarodustat | 0 | -0/059 |  | -0/059 |  |  |  |  |  |
| Daprodustat:ESA | 7 | 0/372 | 0/475 | -0/219 | 0/694 | -0/250 | 1/638 | 0/149 |  |
| Daprodustat:Molidustat | 0 | 0/449 |  | 0/449 |  |  |  |  |  |
| Daprodustat:Placebo | 2 | 0/833 | 0/361 | 1/055 | -0/694 | -1/638 | 0/250 | 0/149 |  |
| Daprodustat:Roxadustat | 0 | 0/144 |  | 0/144 |  |  |  |  |  |
| Daprodustat:Vadadustat | 0 | 0/422 |  | 0/422 |  |  |  |  |  |
| Desidustat:Enarodustat | 0 | 0/258 |  | 0/258 |  |  |  |  |  |
| Desidustat:ESA | 0 | 0/689 |  | 0/689 |  |  |  |  |  |
| Desidustat:Molidustat | 0 | 0/765 |  | 0/765 |  |  |  |  |  |
| Desidustat:Placebo | 1 | 1/150 | 1/150 |  |  |  |  |  |  |
| Desidustat:Roxadustat | 0 | 0/461 |  | 0/461 |  |  |  |  |  |
| Desidustat:Vadadustat | 0 | 0/738 |  | 0/738 |  |  |  |  |  |
| Enarodustat:ESA | 3 | 0/431 | 0/548 | -0/050 | 0/598 | -0/709 | 1/906 | 0/370 |  |
| Enarodustat:Molidustat | 0 | 0/508 |  | 0/508 |  |  |  |  |  |
| Enarodustat:Placebo | 1 | 0/893 | 0/460 | 1/058 | -0/598 | -1/906 | 0/709 | 0/370 |  |
| Enarodustat:Roxadustat | 0 | 0/203 |  | 0/203 |  |  |  |  |  |
| Enarodustat:Vadadustat | 0 | 0/481 |  | 0/481 |  |  |  |  |  |
| Molidustat:ESA | 7 | -0/077 | -0/089 | -0/013 | -0/076 | -0/997 | 0/844 | 0/871 |  |
| Placebo:ESA | 0 | -0/461 |  | -0/461 |  |  |  |  |  |
| Roxadustat:ESA | 10 | 0/228 | 0/142 | 0/597 | -0/454 | -1/166 | 0/257 | 0/211 |  |
| Vadadustat:ESA | 3 | -0/050 | -0/092 | 0/131 | -0/223 | -1/476 | 1/031 | 0/728 |  |
| Molidustat:Placebo | 2 | 0/385 | 0/435 | 0/358 | 0/076 | -0/844 | 0/997 | 0/871 |  |
| Molidustat:Roxadustat | 0 | -0/305 |  | -0/305 |  |  |  |  |  |
| Molidustat:Vadadustat | 0 | -0/027 |  | -0/027 |  |  |  |  |  |
| Placebo:Roxadustat | 6 | -0/690 | -0/841 | -0/387 | -0/454 | -1/166 | 0/257 | 0/211 |  |
| Placebo:Vadadustat | 1 | -0/412 | -0/572 | -0/350 | -0/223 | -1/476 | 1/031 | 0/728 |  |
| Roxadustat:Vadadustat | 0 | 0/278 |  | 0/278 |  |  |  |  |  |

| **sTable 7.** Assessment of inconsistency in the network meta-analysis for Thrombosis | | | | | | | | |
| --- | --- | --- | --- | --- | --- | --- | --- | --- |
| Comparison | No.Studies | NMA | Direct | Indirect | Difference | Diff_95CI_lower | Diff_95CI_upper | pValue |
| Daprodustat:Desidustat | 0 | 0/003 |  | 0/003 |  |  |  |  |
| Daprodustat:Enarodustat | 0 | -0/180 |  | -0/180 |  |  |  |  |
| Daprodustat:ESA | 2 | 0/003 | 0/023 | -0/977 | 1/000 | -2/477 | 4/477 | 0/573 |
| Daprodustat:Placebo | 1 | 0/617 | -0/281 | 0/719 | -1/000 | -4/477 | 2/477 | 0/573 |
| Daprodustat:Roxadustat | 0 | -0/304 |  | -0/304 |  |  |  |  |
| Daprodustat:Vadadustat | 0 | 0/140 |  | 0/140 |  |  |  |  |
| Desidustat:Enarodustat | 0 | -0/182 |  | -0/182 |  |  |  |  |
| Desidustat:ESA | 1 | 4/440E-16 | 0 |  |  |  |  |  |
| Desidustat:Placebo | 0 | 0/615 |  | 0/615 |  |  |  |  |
| Desidustat:Roxadustat | 0 | -0/307 |  | -0/307 |  |  |  |  |
| Desidustat:Vadadustat | 0 | 0/137 |  | 0/137 |  |  |  |  |
| Enarodustat:ESA | 1 | 0/182 | 0/182 |  |  |  |  |  |
| Enarodustat:Placebo | 0 | 0/797 |  | 0/797 |  |  |  |  |
| Enarodustat:Roxadustat | 0 | -0/125 |  | -0/125 |  |  |  |  |
| Enarodustat:Vadadustat | 0 | 0/319 |  | 0/319 |  |  |  |  |
| Placebo:ESA | 0 | -0/615 |  | -0/615 |  |  |  |  |
| Roxadustat:ESA | 4 | 0/307 | 0/287 | 1/286 | -1/000 | -4/477 | 2/477 | 0/573 |
| Vadadustat:ESA | 5 | -0/137 | -0/137 |  |  |  |  |  |
| Placebo:Roxadustat | 2 | -0/922 | -0/982 | 0/018 | -1/000 | -4/477 | 2/477 | 0/573 |
| Placebo:Vadadustat | 0 | -0/478 |  | -0/478 |  |  |  |  |
| Roxadustat:Vadadustat | 0 | 0/444 |  | 0/444 |  |  |  |  |

| **sTable 8.** Assessment of inconsistency in the network meta-analysis for Myocardial Infarction | | | | | | | | |
| --- | --- | --- | --- | --- | --- | --- | --- | --- |
| Comparison | No.Studies | NMA | Direct | Indirect | Difference | Diff_95CI_lower | Diff_95CI_upper | pValue |
| Daprodustat:Desidustat | 0 | 1/022 |  | 1/022 |  |  |  |  |
| Daprodustat:Enarodustat | 0 | -1/178 |  | -1/178 |  |  |  |  |
| Daprodustat:ESA | 5 | -0/080 | -0/080 |  |  |  |  |  |
| Daprodustat:Molidustat | 0 | 0/029 |  | 0/029 |  |  |  |  |
| Daprodustat:Roxadustat | 0 | -0/166 |  | -0/166 |  |  |  |  |
| Daprodustat:Vadadustat | 0 | -0/211 |  | -0/211 |  |  |  |  |
| Desidustat:Enarodustat | 0 | -2/201 |  | -2/201 |  |  |  |  |
| Desidustat:ESA | 1 | -1/102 | -1/102 |  |  |  |  |  |
| Desidustat:Molidustat | 0 | -0/993 |  | -0/993 |  |  |  |  |
| Desidustat:Roxadustat | 0 | -1/189 |  | -1/189 |  |  |  |  |
| Desidustat:Vadadustat | 0 | -1/233 |  | -1/233 |  |  |  |  |
| Enarodustat:ESA | 1 | 1/099 | 1/099 |  |  |  |  |  |
| Enarodustat:Molidustat | 0 | 1/207 |  | 1/207 |  |  |  |  |
| Enarodustat:Roxadustat | 0 | 1/012 |  | 1/012 |  |  |  |  |
| Enarodustat:Vadadustat | 0 | 0/968 |  | 0/968 |  |  |  |  |
| Molidustat:ESA | 4 | -0/109 | -0/109 |  |  |  |  |  |
| Roxadustat:ESA | 6 | 0/087 | 0/087 |  |  |  |  |  |
| Vadadustat:ESA | 3 | 0/131 | 0/131 |  |  |  |  |  |
| Molidustat:Roxadustat | 0 | -0/195 |  | -0/195 |  |  |  |  |
| Molidustat:Vadadustat | 0 | -0/240 |  | -0/240 |  |  |  |  |
| Roxadustat:Vadadustat | 0 | -0/044 |  | -0/044 |  |  |  |  |

| **sTable 9.** Assessment of inconsistency in the network meta-analysis for Stroke | | | | | | | | |
| --- | --- | --- | --- | --- | --- | --- | --- | --- |
| Comparison | No.Studies | NMA | Direct | Indirect | Difference | Diff_95CI_lower | Diff_95CI_upper | pValue |
| Daprodustat:ESA | 3 | 0/021 | 0/021 |  |  |  |  |  |
| Daprodustat:Molidustat | 0 | 0/887 |  | 0/887 |  |  |  |  |
| Daprodustat:Placebo | 0 | 1/426 |  | 1/426 |  |  |  |  |
| Daprodustat:Roxadustat | 0 | 0/690 |  | 0/690 |  |  |  |  |
| Molidustat:ESA | 2 | -0/865 | -0/865 |  |  |  |  |  |
| Placebo:ESA | 0 | -1/405 |  | -1/405 |  |  |  |  |
| Roxadustat:ESA | 1 | -0/669 | -0/669 |  |  |  |  |  |
| Molidustat:Placebo | 0 | 0/540 |  | 0/540 |  |  |  |  |
| Molidustat:Roxadustat | 0 | -0/196 |  | -0/196 |  |  |  |  |
| Placebo:Roxadustat | 1 | -0/736 | -0/736 |  |  |  |  |  |

| **sTable 10.** Assessment of inconsistency in the network meta-analysis for Hypertension | | | | | | | | |
| --- | --- | --- | --- | --- | --- | --- | --- | --- |
| Comparison | No.Studies | NMA | Direct | Indirect | Difference | Diff_95CI_lower | Diff_95CI_upper | pValue |
| Daprodustat:Desidustat | 0 | -0/314 |  | -0/314 |  |  |  |  |
| Daprodustat:Enarodustat | 0 | -0/286 |  | -0/286 |  |  |  |  |
| Daprodustat:ESA | 4 | -0/125 | -0/125 |  |  |  |  |  |
| Daprodustat:Molidustat | 0 | 0/402 |  | 0/402 |  |  |  |  |
| Daprodustat:Placebo | 0 | 0/760 |  | 0/760 |  |  |  |  |
| Daprodustat:Roxadustat | 0 | -0/009 |  | -0/009 |  |  |  |  |
| Daprodustat:Vadadustat | 0 | 0/235 |  | 0/235 |  |  |  |  |
| Desidustat:Enarodustat | 0 | 0/028 |  | 0/028 |  |  |  |  |
| Desidustat:ESA | 2 | 0/189 | 0/189 |  |  |  |  |  |
| Desidustat:Molidustat | 0 | 0/717 |  | 0/717 |  |  |  |  |
| Desidustat:Placebo | 0 | 1/074 |  | 1/074 |  |  |  |  |
| Desidustat:Roxadustat | 0 | 0/305 |  | 0/305 |  |  |  |  |
| Desidustat:Vadadustat | 0 | 0/549 |  | 0/549 |  |  |  |  |
| Enarodustat:ESA | 2 | 0/161 | 0/161 |  |  |  |  |  |
| Enarodustat:Molidustat | 0 | 0/688 |  | 0/688 |  |  |  |  |
| Enarodustat:Placebo | 0 | 1/045 |  | 1/045 |  |  |  |  |
| Enarodustat:Roxadustat | 0 | 0/277 |  | 0/277 |  |  |  |  |
| Enarodustat:Vadadustat | 0 | 0/521 |  | 0/521 |  |  |  |  |
| Molidustat:ESA | 5 | -0/527 | -0/181 | -2/364 | 2/184 | 0/396 | 3/971 | 0/017 |
| Placebo:ESA | 0 | -0/885 |  | -0/885 |  |  |  |  |
| Roxadustat:ESA | 6 | -0/116 | -0/222 | 0/826 | -1/048 | -2/509 | 0/412 | 0/159 |
| Vadadustat:ESA | 3 | -0/360 | -0/423 | 0/324 | -0/747 | -2/722 | 1/228 | 0/458 |
| Molidustat:Placebo | 1 | 0/357 | -1/110 | 1/074 | -2/184 | -3/971 | -0/396 | 0/017 |
| Molidustat:Roxadustat | 0 | -0/412 |  | -0/412 |  |  |  |  |
| Molidustat:Vadadustat | 0 | -0/167 |  | -0/167 |  |  |  |  |
| Placebo:Roxadustat | 3 | -0/769 | -0/980 | 0/068 | -1/048 | -2/509 | 0/412 | 0/159 |
| Placebo:Vadadustat | 1 | -0/525 | -1/109 | -0/362 | -0/747 | -2/722 | 1/228 | 0/458 |
| Roxadustat:Vadadustat | 0 | 0/244 |  | 0/244 |  |  |  |  |

| **sTable 11.** Assessment of inconsistency in the network meta-analysis for Occlusion/Stenosis | | | | | | | | |
| --- | --- | --- | --- | --- | --- | --- | --- | --- |
| Comparison | No.Studies | NMA | Direct | Indirect | Difference | Diff_95CI_lower | Diff_95CI_upper | pValue |
| Daprodustat:Enarodustat | 0 | 0/555 |  | 0/555 |  |  |  |  |
| Daprodustat:ESA | 1 | -0/096 | -0/069 | -1/077 | 1/008 | -3/104 | 5/121 | 0/631 |
| Daprodustat:Molidustat | 0 | -0/017 |  | -0/017 |  |  |  |  |
| Daprodustat:Placebo | 2 | -0/006 | -0/319 | 0/690 | -1/008 | -5/121 | 3/104 | 0/631 |
| Daprodustat:Roxadustat | 0 | -0/717 |  | -0/717 |  |  |  |  |
| Daprodustat:Vadadustat | 0 | -0/014 |  | -0/014 |  |  |  |  |
| Enarodustat:ESA | 1 | -0/651 | -0/651 |  |  |  |  |  |
| Enarodustat:Molidustat | 0 | -0/572 |  | -0/572 |  |  |  |  |
| Enarodustat:Placebo | 0 | -0/561 |  | -0/561 |  |  |  |  |
| Enarodustat:Roxadustat | 0 | -1/271 |  | -1/271 |  |  |  |  |
| Enarodustat:Vadadustat | 0 | -0/569 |  | -0/569 |  |  |  |  |
| Molidustat:ESA | 1 | -0/079 | -0/079 |  |  |  |  |  |
| Placebo:ESA | 0 | -0/090 |  | -0/090 |  |  |  |  |
| Roxadustat:ESA | 3 | 0/621 | 0/621 |  |  |  |  |  |
| Vadadustat:ESA | 2 | -0/082 | -0/181 | 0/828 | -1/008 | -5/121 | 3/104 | 0/631 |
| Molidustat:Placebo | 0 | 0/011 |  | 0/011 |  |  |  |  |
| Molidustat:Roxadustat | 0 | -0/700 |  | -0/700 |  |  |  |  |
| Molidustat:Vadadustat | 0 | 0/003 |  | 0/003 |  |  |  |  |
| Placebo:Roxadustat | 0 | -0/711 |  | -0/711 |  |  |  |  |
| Placebo:Vadadustat | 1 | -0/008 | -0/578 | 0/431 | -1/008 | -5/121 | 3/104 | 0/631 |
| Roxadustat:Vadadustat | 0 | 0/703 |  | 0/703 |  |  |  |  |

| **sTable 12.** Assessment of inconsistency in the network meta-analysis for Transfusion | | | | | | | | |
| --- | --- | --- | --- | --- | --- | --- | --- | --- |
| Comparison | No.Studies | NMA | Direct | Indirect | Difference | Diff_95CI_lower | Diff_95CI_upper | pValue |
| Daprodustat:ESA | 2 | -0/120 | -0/120 |  |  |  |  |  |
| Daprodustat:Molidustat | 0 | -0/268 |  | -0/268 |  |  |  |  |
| Daprodustat:Placebo | 0 | -0/713 |  | -0/713 |  |  |  |  |
| Daprodustat:Roxadustat | 0 | 0/108 |  | 0/108 |  |  |  |  |
| Daprodustat:Vadadustat | 0 | 0/594 |  | 0/594 |  |  |  |  |
| Molidustat:ESA | 4 | 0/148 | 0/034 | 0/980 | -0/946 | -4/178 | 2/285 | 0/566 |
| Placebo:ESA | 0 | 0/592 |  | 0/592 |  |  |  |  |
| Roxadustat:ESA | 4 | -0/228 | -0/223 | -1/169 | 0/946 | -2/285 | 4/178 | 0/566 |
| Vadadustat:ESA | 0 | -0/715 |  | -0/715 |  |  |  |  |
| Molidustat:Placebo | 1 | -0/444 | 0/376 | -0/570 | 0/946 | -2/285 | 4/178 | 0/566 |
| Molidustat:Roxadustat | 0 | 0/377 |  | 0/377 |  |  |  |  |
| Molidustat:Vadadustat | 0 | 0/863 |  | 0/863 |  |  |  |  |
| Placebo:Roxadustat | 4 | 0/821 | 0/827 | -0/120 | 0/946 | -2/285 | 4/178 | 0/566 |
| Placebo:Vadadustat | 3 | 1/307 | 1/307 |  |  |  |  |  |
| Roxadustat:Vadadustat | 0 | 0/486 |  | 0/486 |  |  |  |  |

| **sTable 13.** Assessment of inconsistency in the network meta-analysis for Hyperkalemia | | | | | | | | |
| --- | --- | --- | --- | --- | --- | --- | --- | --- |
| Comparison | No.Studies | NMA | Direct | Indirect | Difference | Diff_95CI_lower | Diff_95CI_upper | pValue |
| Daprodustat:Desidustat | 0 | -1/527 |  | -1/527 |  |  |  |  |
| Daprodustat:ESA | 5 | 0/103 | 0/103 |  |  |  |  |  |
| Daprodustat:Molidustat | 0 | 1/093 |  | 1/093 |  |  |  |  |
| Daprodustat:Placebo | 0 | 0/291 |  | 0/291 |  |  |  |  |
| Daprodustat:Roxadustat | 0 | 0/005 |  | 0/005 |  |  |  |  |
| Daprodustat:Vadadustat | 0 | 0/336 |  | 0/336 |  |  |  |  |
| Desidustat:ESA | 1 | 1/630 | 1/630 |  |  |  |  |  |
| Desidustat:Molidustat | 0 | 2/620 |  | 2/620 |  |  |  |  |
| Desidustat:Placebo | 0 | 1/819 |  | 1/819 |  |  |  |  |
| Desidustat:Roxadustat | 0 | 1/532 |  | 1/532 |  |  |  |  |
| Desidustat:Vadadustat | 0 | 1/863 |  | 1/863 |  |  |  |  |
| Molidustat:ESA | 3 | -0/990 | -0/476 | -2/941 | 2/465 | 0/817 | 4/113 | 0/003 |
| Placebo:ESA | 0 | -0/189 |  | -0/189 |  |  |  |  |
| Roxadustat:ESA | 9 | 0/098 | 0/016 | 1/410 | -1/395 | -2/859 | 0/070 | 0/062 |
| Vadadustat:ESA | 4 | -0/233 | -0/253 | 1/973 | -2/227 | -5/168 | 0/715 | 0/138 |
| Molidustat:Placebo | 1 | -0/801 | -2/569 | -0/104 | -2/465 | -4/113 | -0/817 | 0/003 |
| Molidustat:Roxadustat | 0 | -1/088 |  | -1/088 |  |  |  |  |
| Molidustat:Vadadustat | 0 | -0/757 |  | -0/757 |  |  |  |  |
| Placebo:Roxadustat | 6 | -0/287 | -0/338 | 1/056 | -1/395 | -2/859 | 0/070 | 0/062 |
| Placebo:Vadadustat | 1 | 0/044 | -2/113 | 0/114 | -2/227 | -5/168 | 0/715 | 0/138 |
| Roxadustat:Vadadustat | 0 | 0/331 |  | 0/331 |  |  |  |  |

| **sTable 14.** Assessment of inconsistency in the network meta-analysis for Diabetic Retinopathy | | | | | | | | |
| --- | --- | --- | --- | --- | --- | --- | --- | --- |
| Comparison | No.Studies | NMA | Direct | Indirect | Difference | Diff_95CI_lower | Diff_95CI_upper | pValue |
| Daprodustat:Enarodustat | 0 | -1/076 |  | -1/076 |  |  |  |  |
| Daprodustat:ESA | 2 | 0/022 | 0/022 |  |  |  |  |  |
| Daprodustat:Molidustat | 0 | -0/813 |  | -0/813 |  |  |  |  |
| Daprodustat:Roxadustat | 0 | -1/090 |  | -1/090 |  |  |  |  |
| Daprodustat:Vadadustat | 0 | -0/037 |  | -0/037 |  |  |  |  |
| Enarodustat:ESA | 1 | 1/099 | 1/099 |  |  |  |  |  |
| Enarodustat:Molidustat | 0 | 0/264 |  | 0/264 |  |  |  |  |
| Enarodustat:Roxadustat | 0 | -0/013 |  | -0/013 |  |  |  |  |
| Enarodustat:Vadadustat | 0 | 1/039 |  | 1/039 |  |  |  |  |
| Molidustat:ESA | 3 | 0/835 | 0/835 |  |  |  |  |  |
| Roxadustat:ESA | 1 | 1/112 | 1/112 |  |  |  |  |  |
| Vadadustat:ESA | 4 | 0/059 | 0/059 |  |  |  |  |  |
| Molidustat:Roxadustat | 0 | -0/277 |  | -0/277 |  |  |  |  |
| Molidustat:Vadadustat | 0 | 0/775 |  | 0/775 |  |  |  |  |
| Roxadustat:Vadadustat | 0 | 1/052 |  | 1/052 |  |  |  |  |

| **sTable 15.** Assessment of inconsistency in the network meta-analysis for Headache | | | | | | | | |
| --- | --- | --- | --- | --- | --- | --- | --- | --- |
| Comparison | No.Studies | NMA | Direct | Indirect | Difference | Diff_95CI_lower | Diff_95CI_upper | pValue |
| Daprodustat:Desidustat | 0 | 0/619 |  | 0/619 |  |  |  |  |
| Daprodustat:ESA | 3 | 0/207 | 0/253 | -1/025 | 1/277 | -2/059 | 4/614 | 0/453 |
| Daprodustat:Molidustat | 0 | 0/941 |  | 0/941 |  |  |  |  |
| Daprodustat:Placebo | 1 | 0/379 | -0/829 | 0/448 | -1/277 | -4/614 | 2/059 | 0/453 |
| Daprodustat:Roxadustat | 0 | 0/237 |  | 0/237 |  |  |  |  |
| Daprodustat:Vadadustat | 0 | -0/669 |  | -0/669 |  |  |  |  |
| Desidustat:ESA | 1 | -0/412 | -0/262 | -1/847 | 1/585 | -1/687 | 4/857 | 0/343 |
| Desidustat:Molidustat | 0 | 0/322 |  | 0/322 |  |  |  |  |
| Desidustat:Placebo | 1 | -0/240 | -1/644 | -0/059 | -1/585 | -4/857 | 1/687 | 0/343 |
| Desidustat:Roxadustat | 0 | -0/382 |  | -0/382 |  |  |  |  |
| Desidustat:Vadadustat | 0 | -1/288 |  | -1/288 |  |  |  |  |
| Molidustat:ESA | 1 | -0/735 | -0/735 |  |  |  |  |  |
| Placebo:ESA | 0 | -0/172 |  | -0/172 |  |  |  |  |
| Roxadustat:ESA | 4 | -0/030 | -0/055 | 0/837 | -0/892 | -2/434 | 0/649 | 0/256 |
| Vadadustat:ESA | 1 | 0/876 | 1/001 | 0/571 | 0/431 | -1/522 | 2/383 | 0/666 |
| Molidustat:Placebo | 0 | -0/562 |  | -0/562 |  |  |  |  |
| Molidustat:Roxadustat | 0 | -0/704 |  | -0/704 |  |  |  |  |
| Molidustat:Vadadustat | 0 | -1/610 |  | -1/610 |  |  |  |  |
| Placebo:Roxadustat | 3 | -0/142 | -0/201 | 0/692 | -0/892 | -2/434 | 0/649 | 0/256 |
| Placebo:Vadadustat | 1 | -1/048 | -0/767 | -1/198 | 0/431 | -1/522 | 2/383 | 0/666 |
| Roxadustat:Vadadustat | 0 | -0/906 |  | -0/906 |  |  |  |  |

| **sTable 16.** Assessment of inconsistency in the network meta-analysis for Nausea | | | | | | | | |
| --- | --- | --- | --- | --- | --- | --- | --- | --- |
| Comparison | No.Studies | NMA | Direct | Indirect | Difference | Diff_95CI_lower | Diff_95CI_upper | pValue |
| Daprodustat:Desidustat | 0 | -0/779 |  | -0/779 |  |  |  |  |
| Daprodustat:ESA | 3 | 0/089 | 0/181 | -0/534 | 0/716 | -1/787 | 3/219 | 0/575 |
| Daprodustat:Molidustat | 0 | 0/604 |  | 0/604 |  |  |  |  |
| Daprodustat:Placebo | 1 | 0/551 | -0/020 | 0/695 | -0/716 | -3/219 | 1/787 | 0/575 |
| Daprodustat:Roxadustat | 0 | -0/157 |  | -0/157 |  |  |  |  |
| Daprodustat:Vadadustat | 0 | -0/196 |  | -0/196 |  |  |  |  |
| Desidustat:ESA | 1 | 0/868 | 0/868 |  |  |  |  |  |
| Desidustat:Molidustat | 0 | 1/383 |  | 1/383 |  |  |  |  |
| Desidustat:Placebo | 0 | 1/330 |  | 1/330 |  |  |  |  |
| Desidustat:Roxadustat | 0 | 0/622 |  | 0/622 |  |  |  |  |
| Desidustat:Vadadustat | 0 | 0/584 |  | 0/584 |  |  |  |  |
| Molidustat:ESA | 3 | -0/515 | -0/515 |  |  |  |  |  |
| Placebo:ESA | 0 | -0/462 |  | -0/462 |  |  |  |  |
| Roxadustat:ESA | 4 | 0/246 | 0/244 | 0/259 | -0/015 | -1/475 | 1/444 | 0/984 |
| Vadadustat:ESA | 3 | 0/285 | 0/266 | 0/549 | -0/283 | -1/908 | 1/343 | 0/733 |
| Molidustat:Placebo | 0 | -0/053 |  | -0/053 |  |  |  |  |
| Molidustat:Roxadustat | 0 | -0/761 |  | -0/761 |  |  |  |  |
| Molidustat:Vadadustat | 0 | -0/799 |  | -0/799 |  |  |  |  |
| Placebo:Roxadustat | 3 | -0/708 | -0/710 | -0/695 | -0/015 | -1/475 | 1/444 | 0/984 |
| Placebo:Vadadustat | 1 | -0/747 | -0/954 | -0/671 | -0/283 | -1/908 | 1/343 | 0/733 |
| Roxadustat:Vadadustat | 0 | -0/039 |  | -0/039 |  |  |  |  |

| **sTable 17.** Assessment of inconsistency in the network meta-analysis for Vomiting | | | | | | | | |
| --- | --- | --- | --- | --- | --- | --- | --- | --- |
| Comparison | No.Studies | NMA | Direct | Indirect | Difference | Diff_95CI_lower | Diff_95CI_upper | pValue |
| Daprodustat:Desidustat | 0 | 0/183 |  | 0/183 |  |  |  |  |
| Daprodustat:Enarodustat | 0 | -1/265 |  | -1/265 |  |  |  |  |
| Daprodustat:ESA | 1 | 0/313 | 0/335 | 0/088 | 0/247 | -3/073 | 3/566 | 0/884 |
| Daprodustat:Molidustat | 0 | 0/210 |  | 0/210 |  |  |  |  |
| Daprodustat:Placebo | 1 | 0/247 | 0/038 | 0/284 | -0/247 | -3/566 | 3/073 | 0/884 |
| Daprodustat:Roxadustat | 0 | -0/036 |  | -0/036 |  |  |  |  |
| Daprodustat:Vadadustat | 0 | 0/311 |  | 0/311 |  |  |  |  |
| Desidustat:Enarodustat | 0 | -1/448 |  | -1/448 |  |  |  |  |
| Desidustat:ESA | 2 | 0/130 | -0/003 | 0/909 | -0/912 | -3/786 | 1/961 | 0/534 |
| Desidustat:Molidustat | 0 | 0/028 |  | 0/028 |  |  |  |  |
| Desidustat:Placebo | 1 | 0/064 | 0/765 | -0/148 | 0/912 | -1/961 | 3/786 | 0/534 |
| Desidustat:Roxadustat | 0 | -0/218 |  | -0/218 |  |  |  |  |
| Desidustat:Vadadustat | 0 | 0/129 |  | 0/129 |  |  |  |  |
| Enarodustat:ESA | 1 | 1/578 | 1/578 |  |  |  |  |  |
| Enarodustat:Molidustat | 0 | 1/475 |  | 1/475 |  |  |  |  |
| Enarodustat:Placebo | 0 | 1/512 |  | 1/512 |  |  |  |  |
| Enarodustat:Roxadustat | 0 | 1/229 |  | 1/229 |  |  |  |  |
| Enarodustat:Vadadustat | 0 | 1/577 |  | 1/577 |  |  |  |  |
| Molidustat:ESA | 2 | 0/103 | 0/254 | -1/004 | 1/258 | -1/565 | 4/081 | 0/383 |
| Placebo:ESA | 0 | 0/066 |  | 0/066 |  |  |  |  |
| Roxadustat:ESA | 6 | 0/349 | 0/330 | 0/579 | -0/249 | -2/140 | 1/642 | 0/796 |
| Vadadustat:ESA | 3 | 0/002 | 0/002 |  |  |  |  |  |
| Molidustat:Placebo | 1 | 0/037 | -0/958 | 0/300 | -1/258 | -4/081 | 1/565 | 0/383 |
| Molidustat:Roxadustat | 0 | -0/246 |  | -0/246 |  |  |  |  |
| Molidustat:Vadadustat | 0 | 0/101 |  | 0/101 |  |  |  |  |
| Placebo:Roxadustat | 1 | -0/283 | -0/323 | -0/074 | -0/249 | -2/140 | 1/642 | 0/796 |
| Placebo:Vadadustat | 0 | 0/064 |  | 0/064 |  |  |  |  |
| Roxadustat:Vadadustat | 0 | 0/347 |  | 0/347 |  |  |  |  |

| **sTable 18.** Assessment of inconsistency in the network meta-analysis for Diarrhea | | | | | | | | | |
| --- | --- | --- | --- | --- | --- | --- | --- | --- | --- |
| Comparison | No.Studies | NMA | Direct | Indirect | Difference | Diff_95CI_lower | Diff_95CI_upper | pValue |  |
| Daprodustat:Desidustat | 0 | 0/427 |  | 0/427 |  |  |  |  |  |
| Daprodustat:Enarodustat | 0 | 1/337 |  | 1/337 |  |  |  |  |  |
| Daprodustat:ESA | 5 | 0/199 | 0/218 | -0/313 | 0/532 | -1/754 | 2/817 | 0/648 |  |
| Daprodustat:Molidustat | 0 | 0/290 |  | 0/290 |  |  |  |  |  |
| Daprodustat:Placebo | 2 | 0/390 | -0/105 | 0/427 | -0/532 | -2/817 | 1/754 | 0/648 |  |
| Daprodustat:Roxadustat | 0 | 0/002 |  | 0/002 |  |  |  |  |  |
| Daprodustat:Vadadustat | 0 | -0/112 |  | -0/112 |  |  |  |  |  |
| Desidustat:Enarodustat | 0 | 0/909 |  | 0/909 |  |  |  |  |  |
| Desidustat:ESA | 1 | -0/228 | -0/228 |  |  |  |  |  |  |
| Desidustat:Molidustat | 0 | -0/138 |  | -0/138 |  |  |  |  |  |
| Desidustat:Placebo | 0 | -0/038 |  | -0/038 |  |  |  |  |  |
| Desidustat:Roxadustat | 0 | -0/425 |  | -0/425 |  |  |  |  |  |
| Desidustat:Vadadustat | 0 | -0/539 |  | -0/539 |  |  |  |  |  |
| Enarodustat:ESA | 1 | -1/138 | -1/138 |  |  |  |  |  |  |
| Enarodustat:Molidustat | 0 | -1/047 |  | -1/047 |  |  |  |  |  |
| Enarodustat:Placebo | 0 | -0/947 |  | -0/947 |  |  |  |  |  |
| Enarodustat:Roxadustat | 0 | -1/334 |  | -1/334 |  |  |  |  |  |
| Enarodustat:Vadadustat | 0 | -1/449 |  | -1/449 |  |  |  |  |  |
| Molidustat:ESA | 6 | -0/091 | -0/075 | -0/447 | 0/372 | -1/963 | 2/707 | 0/755 |  |
| Placebo:ESA | 0 | -0/191 |  | -0/191 |  |  |  |  |  |
| Roxadustat:ESA | 2 | 0/196 | 0/203 | 0/075 | 0/128 | -0/974 | 1/230 | 0/820 |  |
| Vadadustat:ESA | 4 | 0/311 | 0/304 | 0/815 | -0/511 | -1/872 | 0/850 | 0/462 |  |
| Molidustat:Placebo | 1 | 0/100 | -0/244 | 0/128 | -0/372 | -2/707 | 1/963 | 0/755 |  |
| Molidustat:Roxadustat | 0 | -0/287 |  | -0/287 |  |  |  |  |  |
| Molidustat:Vadadustat | 0 | -0/402 |  | -0/402 |  |  |  |  |  |
| Placebo:Roxadustat | 5 | -0/387 | -0/373 | -0/501 | 0/128 | -0/974 | 1/230 | 0/820 |  |
| Placebo:Vadadustat | 1 | -0/502 | -0/954 | -0/444 | -0/511 | -1/872 | 0/850 | 0/462 |  |
| Roxadustat:Vadadustat | 0 | -0/114 |  | -0/114 |  |  |  |  |  |

| **sTable 19.** Assessment of inconsistency in the network meta-analysis for Constipation | | | | | | | | |
| --- | --- | --- | --- | --- | --- | --- | --- | --- |
| Comparison | No.Studies | NMA | Direct | Indirect | Difference | Diff_95CI_lower | Diff_95CI_upper | pValue |
| Daprodustat:ESA | 2 | -0/306 | -0/306 |  |  |  |  |  |
| Daprodustat:Molidustat | 0 | -0/350 |  | -0/350 |  |  |  |  |
| Daprodustat:Placebo | 0 | 0/055 |  | 0/055 |  |  |  |  |
| Daprodustat:Roxadustat | 0 | -0/389 |  | -0/389 |  |  |  |  |
| Daprodustat:Vadadustat | 0 | -0/092 |  | -0/092 |  |  |  |  |
| Molidustat:ESA | 3 | 0/044 | 0/074 | -0/392 | 0/465 | -1/867 | 2/798 | 0/696 |
| Placebo:ESA | 0 | -0/361 |  | -0/361 |  |  |  |  |
| Roxadustat:ESA | 2 | 0/083 | 0/045 | 0/794 | -0/748 | -2/094 | 0/597 | 0/276 |
| Vadadustat:ESA | 2 | -0/214 | -0/086 | -0/890 | 0/804 | -0/762 | 2/370 | 0/314 |
| Molidustat:Placebo | 1 | 0/405 | -0/010 | 0/455 | -0/465 | -2/798 | 1/867 | 0/696 |
| Molidustat:Roxadustat | 0 | -0/039 |  | -0/039 |  |  |  |  |
| Molidustat:Vadadustat | 0 | 0/258 |  | 0/258 |  |  |  |  |
| Placebo:Roxadustat | 1 | -0/444 | -0/515 | 0/234 | -0/748 | -2/094 | 0/597 | 0/276 |
| Placebo:Vadadustat | 1 | -0/147 | 0/448 | -0/356 | 0/804 | -0/762 | 2/370 | 0/314 |
| Roxadustat:Vadadustat | 0 | 0/297 |  | 0/297 |  |  |  |  |

| **sTable 20.** Assessment of inconsistency in the network meta-analysis for Peripheral Edema | | | | | | | | |
| --- | --- | --- | --- | --- | --- | --- | --- | --- |
| Comparison | No.Studies | NMA | Direct | Indirect | Difference | Diff_95CI_lower | Diff_95CI_upper | pValue |
| Daprodustat:Desidustat | 0 | -0/478 |  | -0/478 |  |  |  |  |
| Daprodustat:Enarodustat | 0 | -0/918 |  | -0/918 |  |  |  |  |
| Daprodustat:ESA | 4 | 0/195 | 0/195 |  |  |  |  |  |
| Daprodustat:Molidustat | 0 | -0/864 |  | -0/864 |  |  |  |  |
| Daprodustat:Placebo | 0 | 0/176 |  | 0/176 |  |  |  |  |
| Daprodustat:Roxadustat | 0 | -0/156 |  | -0/156 |  |  |  |  |
| Daprodustat:Vadadustat | 0 | 0/082 |  | 0/082 |  |  |  |  |
| Desidustat:Enarodustat | 0 | -0/440 |  | -0/440 |  |  |  |  |
| Desidustat:ESA | 2 | 0/673 | 0/673 |  |  |  |  |  |
| Desidustat:Molidustat | 0 | -0/386 |  | -0/386 |  |  |  |  |
| Desidustat:Placebo | 0 | 0/653 |  | 0/653 |  |  |  |  |
| Desidustat:Roxadustat | 0 | 0/321 |  | 0/321 |  |  |  |  |
| Desidustat:Vadadustat | 0 | 0/560 |  | 0/560 |  |  |  |  |
| Enarodustat:ESA | 2 | 1/113 | 1/113 |  |  |  |  |  |
| Enarodustat:Molidustat | 0 | 0/054 |  | 0/054 |  |  |  |  |
| Enarodustat:Placebo | 0 | 1/093 |  | 1/093 |  |  |  |  |
| Enarodustat:Roxadustat | 0 | 0/761 |  | 0/761 |  |  |  |  |
| Enarodustat:Vadadustat | 0 | 1/000 |  | 1/000 |  |  |  |  |
| Molidustat:ESA | 2 | 1/059 | 1/059 |  |  |  |  |  |
| Placebo:ESA | 0 | 0/019 |  | 0/019 |  |  |  |  |
| Roxadustat:ESA | 4 | 0/351 | 0/111 | 0/803 | -0/691 | -1/997 | 0/615 | 0/299 |
| Vadadustat:ESA | 3 | 0/112 | 0/130 | -0/561 | 0/691 | -0/615 | 1/997 | 0/299 |
| Molidustat:Placebo | 0 | 1/040 |  | 1/040 |  |  |  |  |
| Molidustat:Roxadustat | 0 | 0/708 |  | 0/708 |  |  |  |  |
| Molidustat:Vadadustat | 0 | 0/946 |  | 0/946 |  |  |  |  |
| Placebo:Roxadustat | 5 | -0/332 | -0/351 | 0/340 | -0/691 | -1/997 | 0/615 | 0/299 |
| Placebo:Vadadustat | 1 | -0/093 | 0/321 | -0/370 | 0/691 | -0/615 | 1/997 | 0/299 |
| Roxadustat:Vadadustat | 0 | 0/239 |  | 0/239 |  |  |  |  |


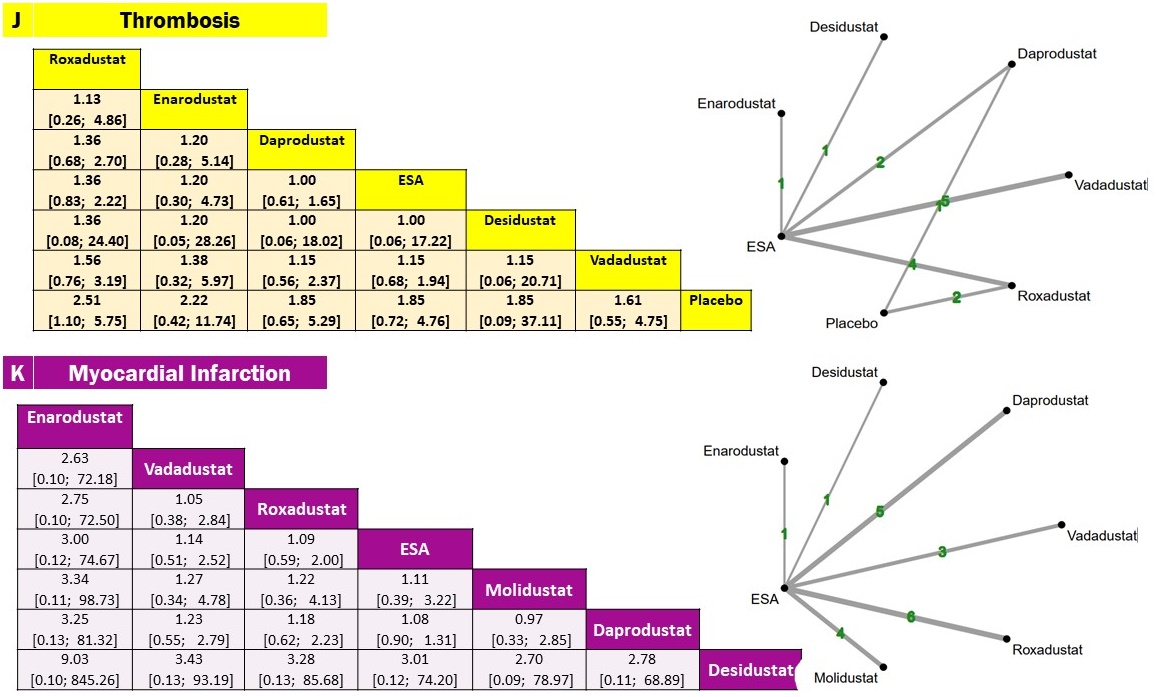


**sFigure 1.** *Network meta-analysis results for two adverse events: (J) Thrombosis and (K) Myocardial Infarction. For each outcome, the league table displays pairwise comparisons of treatments (left), and the network plot illustrates the geometry of direct comparisons (right).*


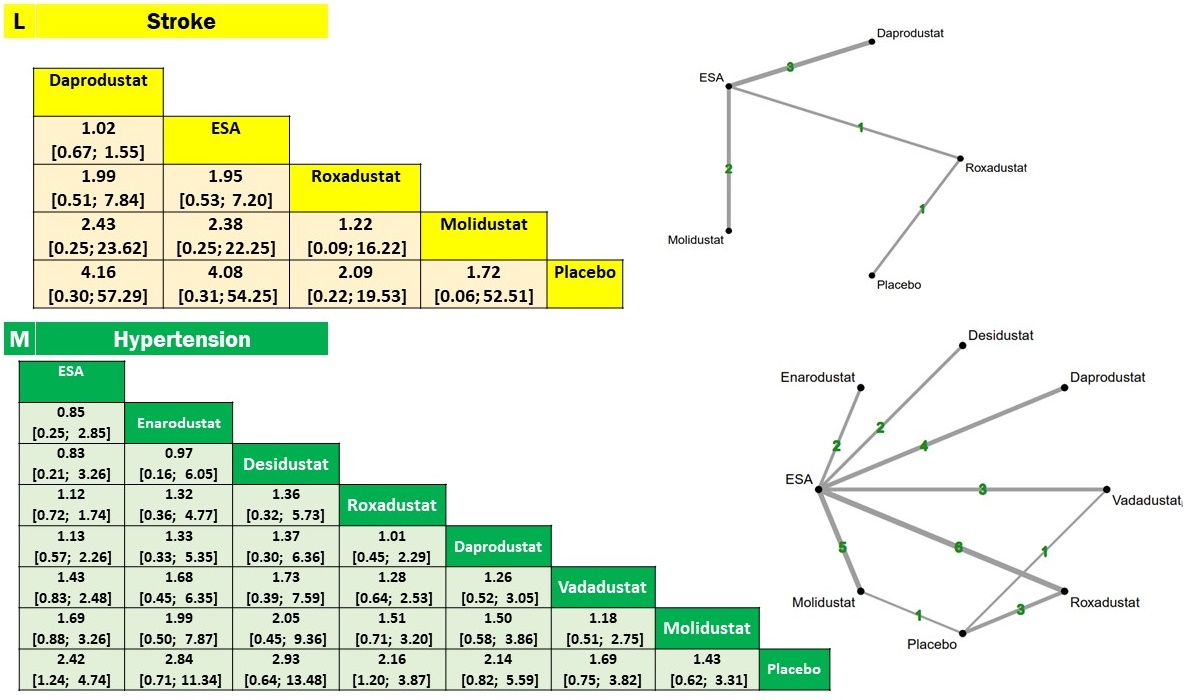


**sFigure 2.** *Network meta-analysis results for two adverse events: (L) Stroke and (M) Hypertension. For each outcome, the league table displays pairwise comparisons of treatments (left), and the network plot illustrates the geometry of direct comparisons (right).*


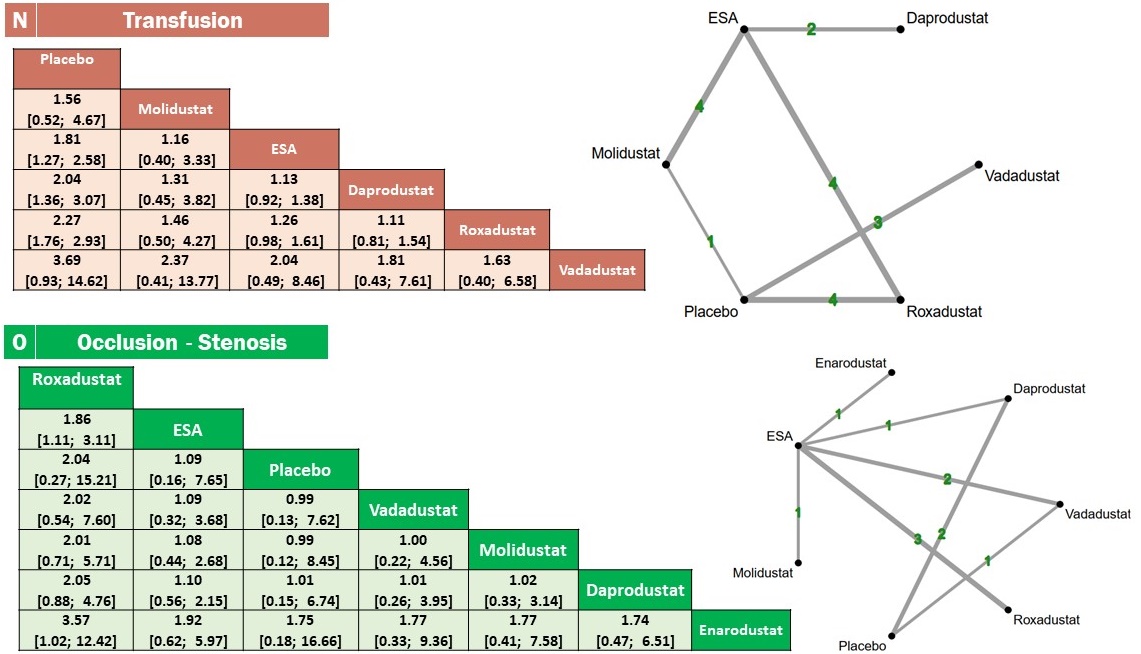


**sFigure 3.** *Network meta-analysis results for two adverse events: (N) Transfuison and (O) Occlusion/Stenosis. For each outcome, the league table displays pairwise comparisons of treatments (left), and the network plot illustrates the geometry of direct comparisons (right).*


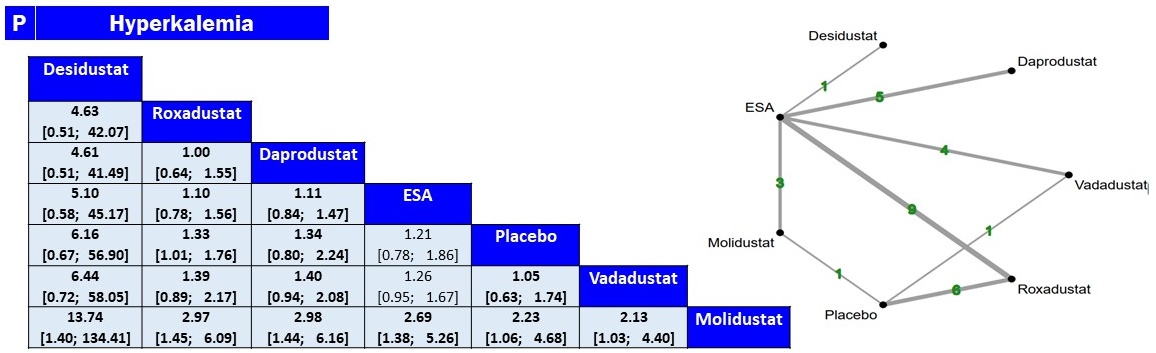


**sFigure 4.** *Network meta-analysis results for one adverse event: (P) Hyperkalemia, the league table displays pairwise comparisons of treatments (left), and the network plot illustrates the geometry of direct comparisons (right).*


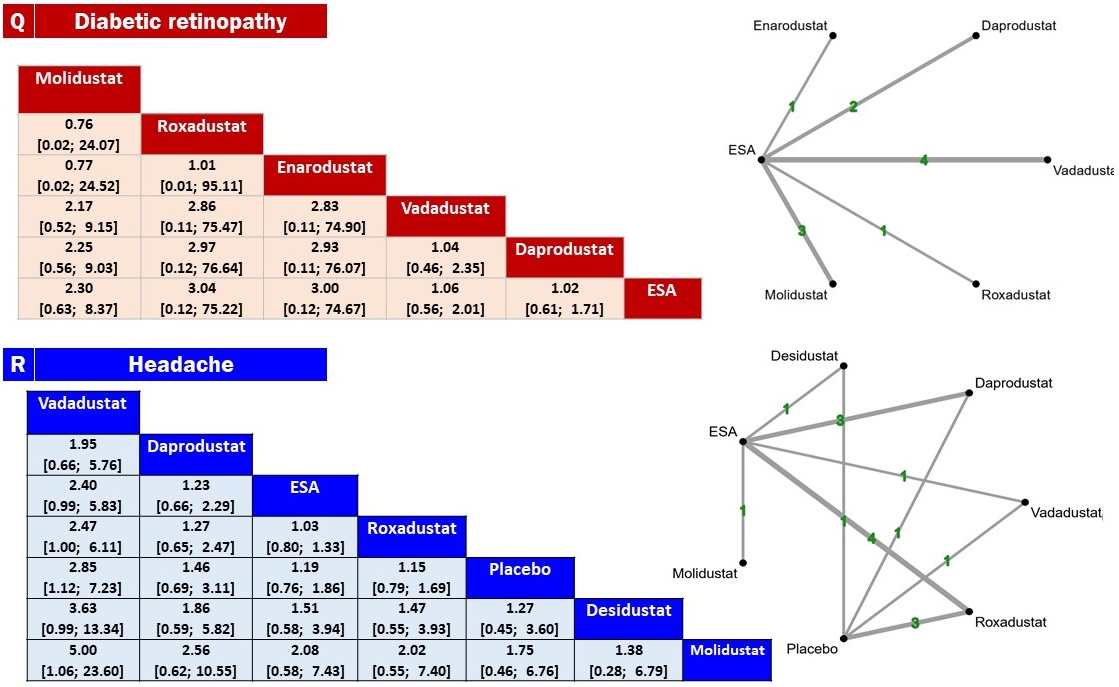


**sFigure 5.** *Network meta-analysis results for two adverse events: (Q) Diabetic retinopathy and (R)Headache. For each outcome, the league table displays pairwise comparisons of treatments (left), and the network plot illustrates the geometry of direct comparisons (right).*


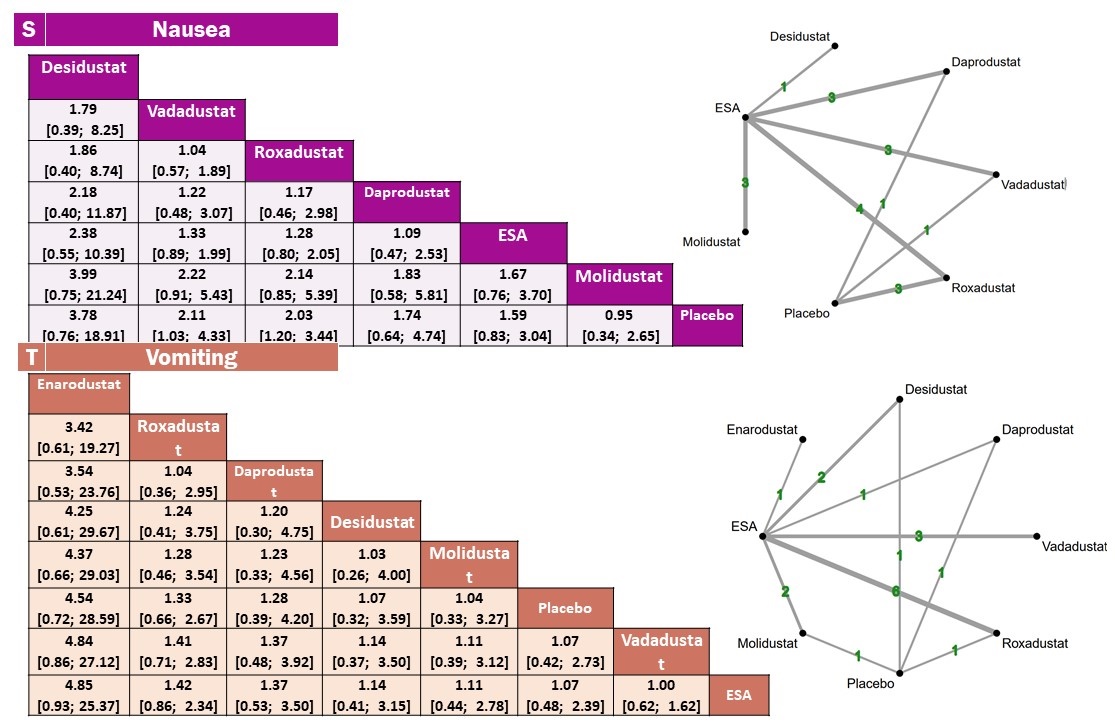


**sFigure 6.** *Network meta-analysis results for two adverse events: (S) Nausea and (T)Vomiting. For each outcome, the league table displays pairwise comparisons of treatments (left), and the network plot illustrates the geometry of direct comparisons (right).*


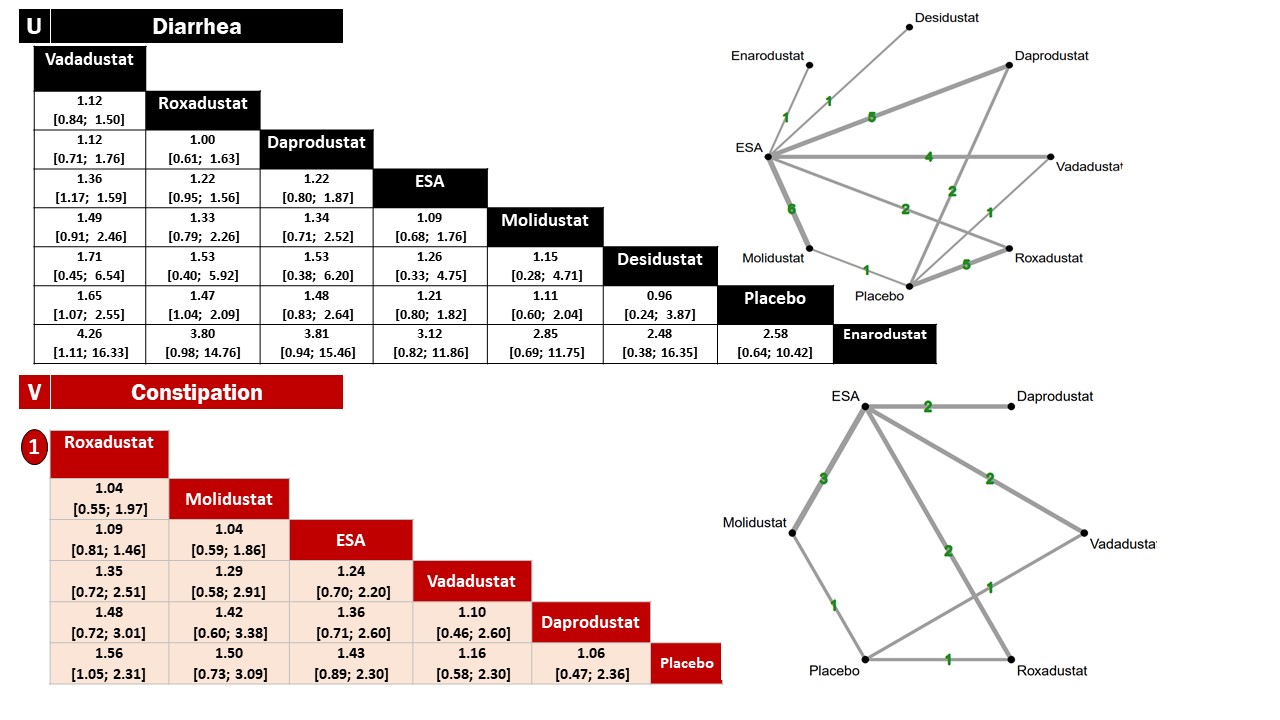


**sFigure 7.** *Network meta-analysis results for two adverse events: (U) Diarrhea and (V)Constipation. For each outcome, the league table displays pairwise comparisons of treatments (left), and the network plot illustrates the geometry of direct comparisons (right).*

*
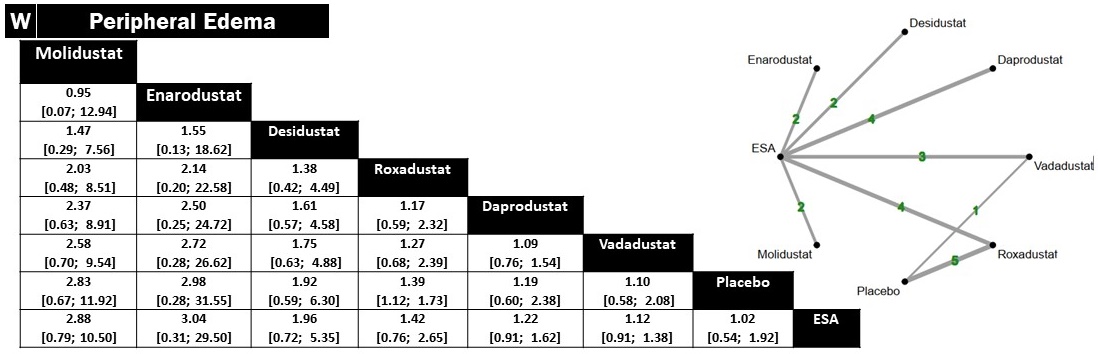
*

**sFigure 8.** *Network meta-analysis results for one adverse event: (w) Peripheral Edema. For each outcome, the league table displays pairwise comparisons of treatments (left), and the network plot illustrates the geometry of direct comparisons (right).*


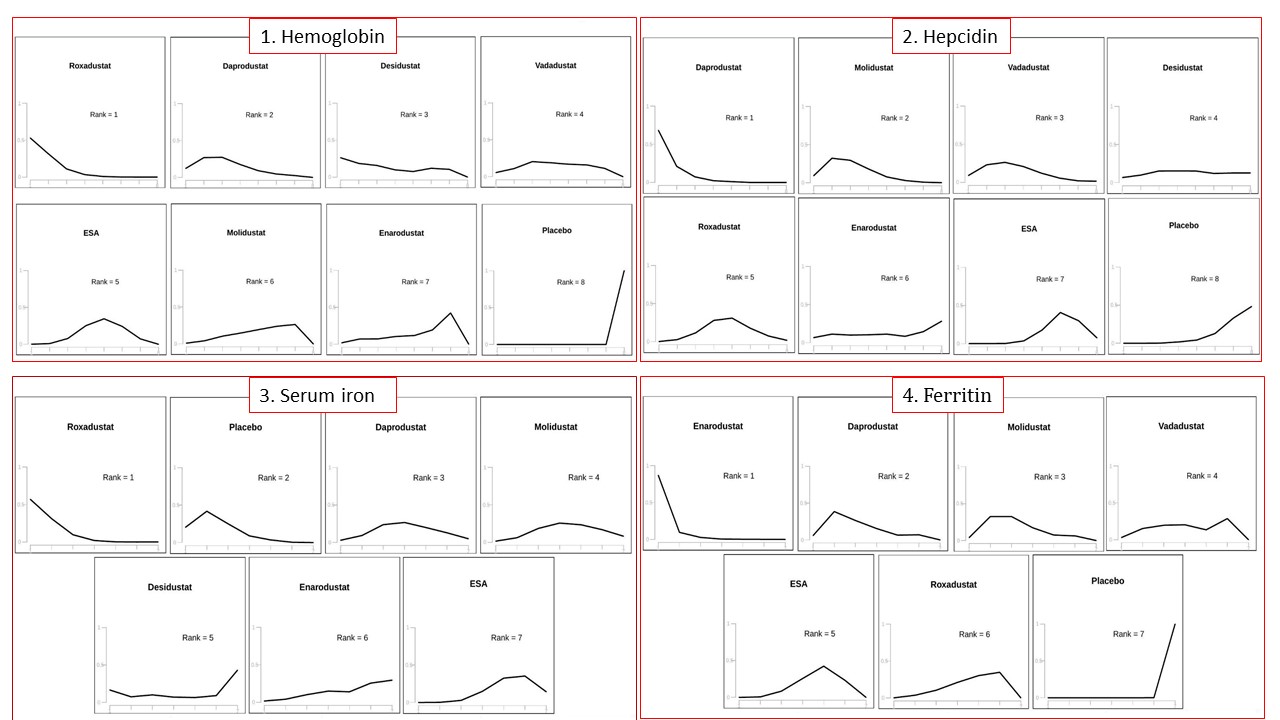


**sFigure 9**. Ranking of treatments based on cumulative probability for four laboratory outcomes: (1) Hemoglobin, (2) Hepcidin, (3) Serum iron, and (4) Ferritin. Rankings are presented for the overall population without stratification by dialysis status. Lower rank values indicate higher effectiveness.


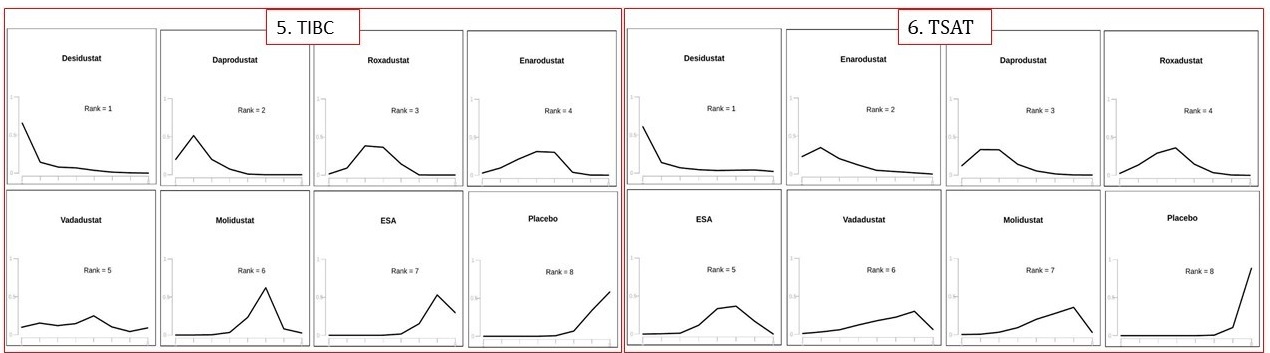


**sFigure 10.** *Ranking of treatments based on cumulative probability for four laboratory outcomes: (5) Total iron-binding capacity (TIBC), (6) Transferrin saturation. Rankings are presented for the overall population without stratification by dialysis status. Lower rank values indicate higher effectiveness.*


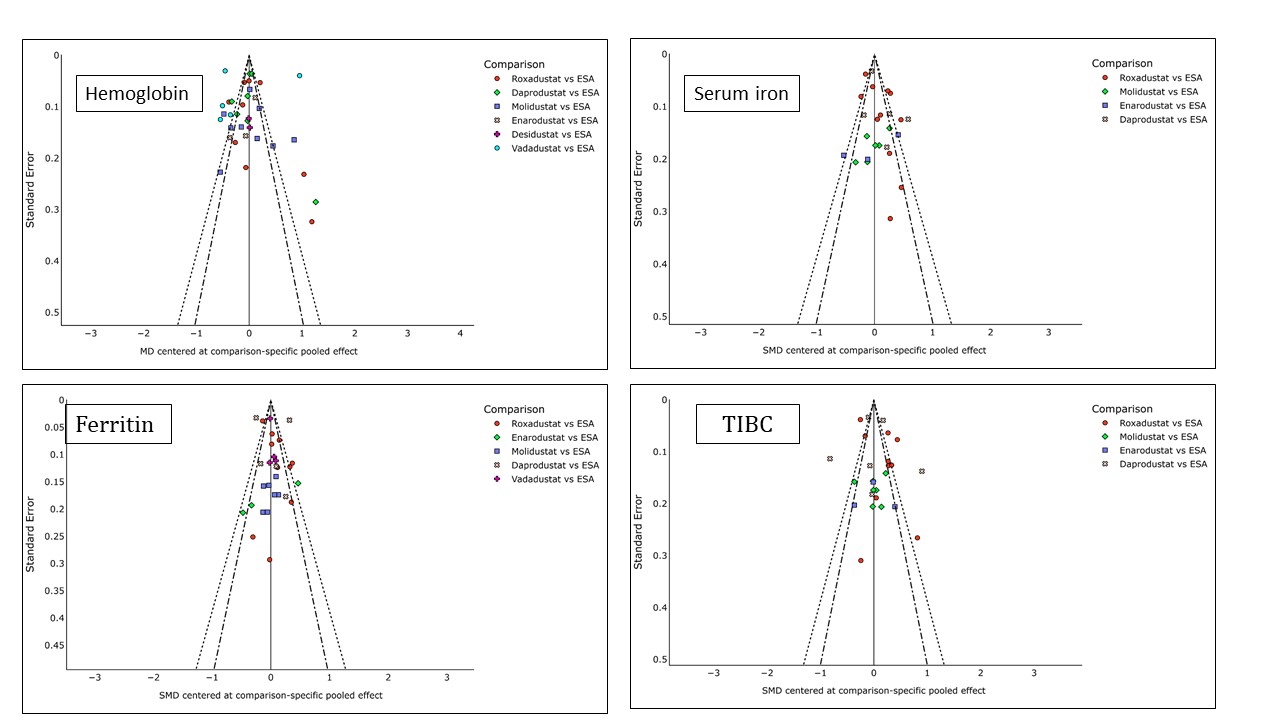


**sFigure 11**. Funnel plots for the assessment of potential publication bias across studies reporting (1) Hemoglobin, (2) Serum iron, (3) Ferritin, and (4) TIBC. Each dot represents an individual study comparison against ESA, and the vertical line indicates the pooled effect size. Symmetry of the plots suggests lower risk of publication bias.


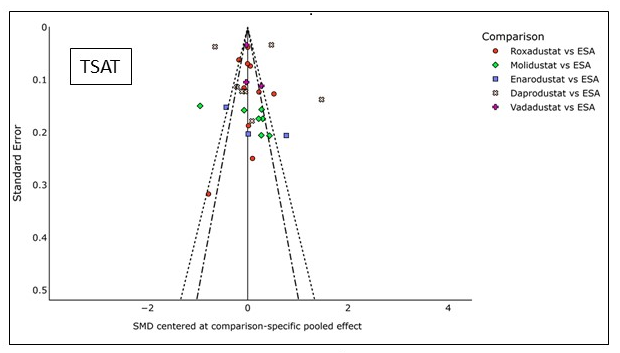


**sFigure 12**. Funnel plots evaluating potential publication bias for studies reporting (5) TSAT. Each dot represents a treatment comparison versus ESA. Symmetrical distributions indicate lower likelihood of publication bias.
